# Non-Pneumatic Anti-Shock Garment (NASG), a First-Aid Device to Decrease Maternal Mortality from Obstetric Hemorrhage: A Cluster Randomized Trial

**DOI:** 10.1371/journal.pone.0076477

**Published:** 2013-10-23

**Authors:** Suellen Miller, Eduardo F. Bergel, Alison M. El Ayadi, Luz Gibbons, Elizabeth A. Butrick, Thulani Magwali, Gricelia Mkumba, Christine Kaseba, N. T. My Huong, Jillian D. Geissler, Mario Merialdi

**Affiliations:** 1 Department of Obstetrics, Gynecology, and Reproductive Sciences, University of California San Francisco, San Francisco, California, United States of America; 2 Instituto de Efectividad Clínica y Sanitaria, Buenos Aires, Argentina; 3 Department of Obstetrics and Gynecology, University of Zimbabwe, Harare, Zimbabwe; 4 Department of Obstetrics and Gynecology, University Teaching Hospital, Lusaka, Zambia; 5 The Department of Reproductive Health and Research of the United Nations Development Programme/United Nations Population Fund/United Nations Children’s Fund/World Health Organization/World Bank Special Programme of Research, Development and Research Training in Human Reproduction, World Health Organization, Geneva, Switzerland; Assiut University Hospital, Egypt

## Abstract

**Background:**

Obstetric hemorrhage is the leading cause of maternal mortality. Using a cluster randomized design, we investigated whether application of the Non-pneumatic Anti-Shock Garment (NASG) before transport to referral hospitals (RHs) from primary health care centers (PHCs) decreased adverse outcomes among women with hypovolemic shock. We hypothesized the NASG group would have a 50% reduction in adverse outcomes.

**Methods and Findings:**

We randomly assigned 38 PHCs in Zambia and Zimbabwe to standard obstetric hemorrhage/shock protocols or the same protocols plus NASG prior to transport. All women received the NASG at the RH. The primary outcomes were maternal mortality; severe, end-organ failure maternal morbidity; and a composite mortality/morbidity outcome, which we labeled extreme adverse outcome (EAO). We also examined whether the NASG contributed to negative side effects and secondary outcomes. The sample size for statistical power was not reached; of a planned 2400 women, 880 were enrolled, 405 in the intervention group. The intervention was associated with a non-significant 46% reduced odds of mortality (OR 0.54, 95% CI 0.14–2.05, p = 0.37) and 54% reduction in composite EAO (OR 0.46, 95% CI 0.13–1.62, p = 0.22). Women with NASGs recovered from shock significantly faster (HR 1.25, 95% CI 1.02–1.52, p = 0.03). No differences were observed in secondary outcomes or negative effects. The main limitation was small sample size.

**Conclusions:**

Despite a lack of statistical significance, the 54% reduced odds of EAO and the significantly faster shock recovery suggest there might be treatment benefits from earlier application of the NASG for women experiencing delays obtaining definitive treatment for hypovolemic shock. As there are no other tools for shock management outside of referral facilities, and no safety issues found, consideration of NASGs as a temporizing measure during delays may be warranted. A pragmatic study with rigorous evaluation is suggested for further research.

**Trial Registration:**

ClinicalTrials.gov NCT00488462

## Introduction

Obstetric hemorrhage (OH) is the leading cause of maternal mortality, responsible for 25–50% of maternal deaths [1]. Uncontrolled hemorrhage can lead to irreversible hypovolemic shock, multiple organ dysfunction syndrome, and mortality. Current obstetric and midwifery guidelines stress the use of uterotonics (oxytocin, misoprostol) to prevent postpartum hemorrhage (PPH) due to uterine atony [2], [3]; however, even under randomized trial conditions, uterotonics can only reduce PPH by 24% to 60% [4], [5]. Uterotonics are also recommended for treatment of atonic PPH, but they do not always stop hemorrhage. Additional means of stopping atonic hemorrhage, such as balloon tamponade, are currently being recommended [2], but not all OH is due to atonic etiologies. Neither administration of uterotonics nor balloon tamponade will treat non-atonic OH (e.g. ruptured uterus, ruptured ectopic pregnancy, vaginal/perineal lacerations, etc). Finally, if a woman bleeding from any OH etiology has lost so much blood that she has gone into shock, even if bleeding can be controlled, the woman may still need blood transfusions.

Blood transfusions and surgery are sometimes the only definitive treatment for severe OH and hypovolemic shock, but they are frequently only available at the highest level of the health system. For most women, accessing such care therefore relies on overcoming a series of delays that are associated with high rates of maternal death in limited-resource settings: recognizing complications, deciding to seek care, finding transport to care, and receiving quality comprehensive emergency obstetric care at referral facilities [6], [7]. Until recently, the only tools available at lower levels might be elevating the woman’s lower extremities and referral. Transport during referral can take hours, sometimes days. Upon arrival at a tertiary center (or even for women who begin hemorrhage in a tertiary facility), there may be long delays before blood transfusions can be arranged and completed.

**Figure 1:**
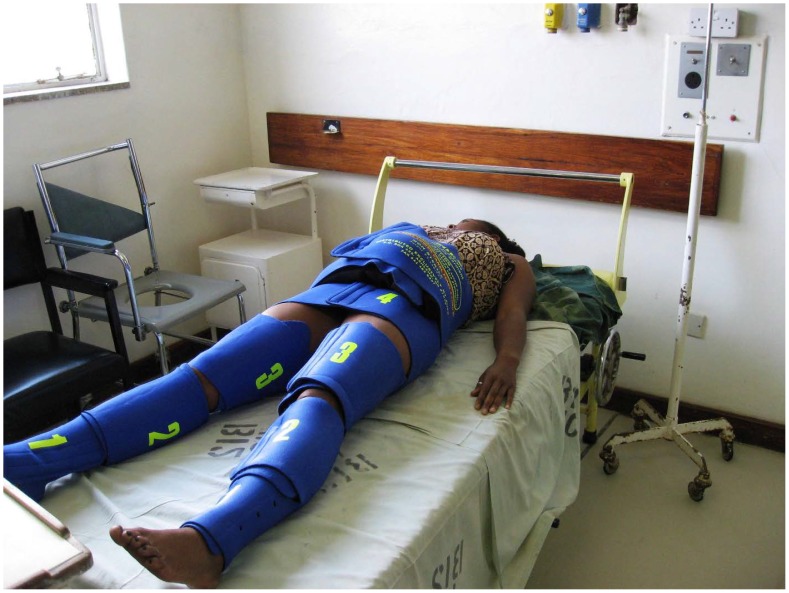
Non-pneumatic Anti-Shock Garment (NASG) Photo. A model in an NASG.

**Figure 2:**
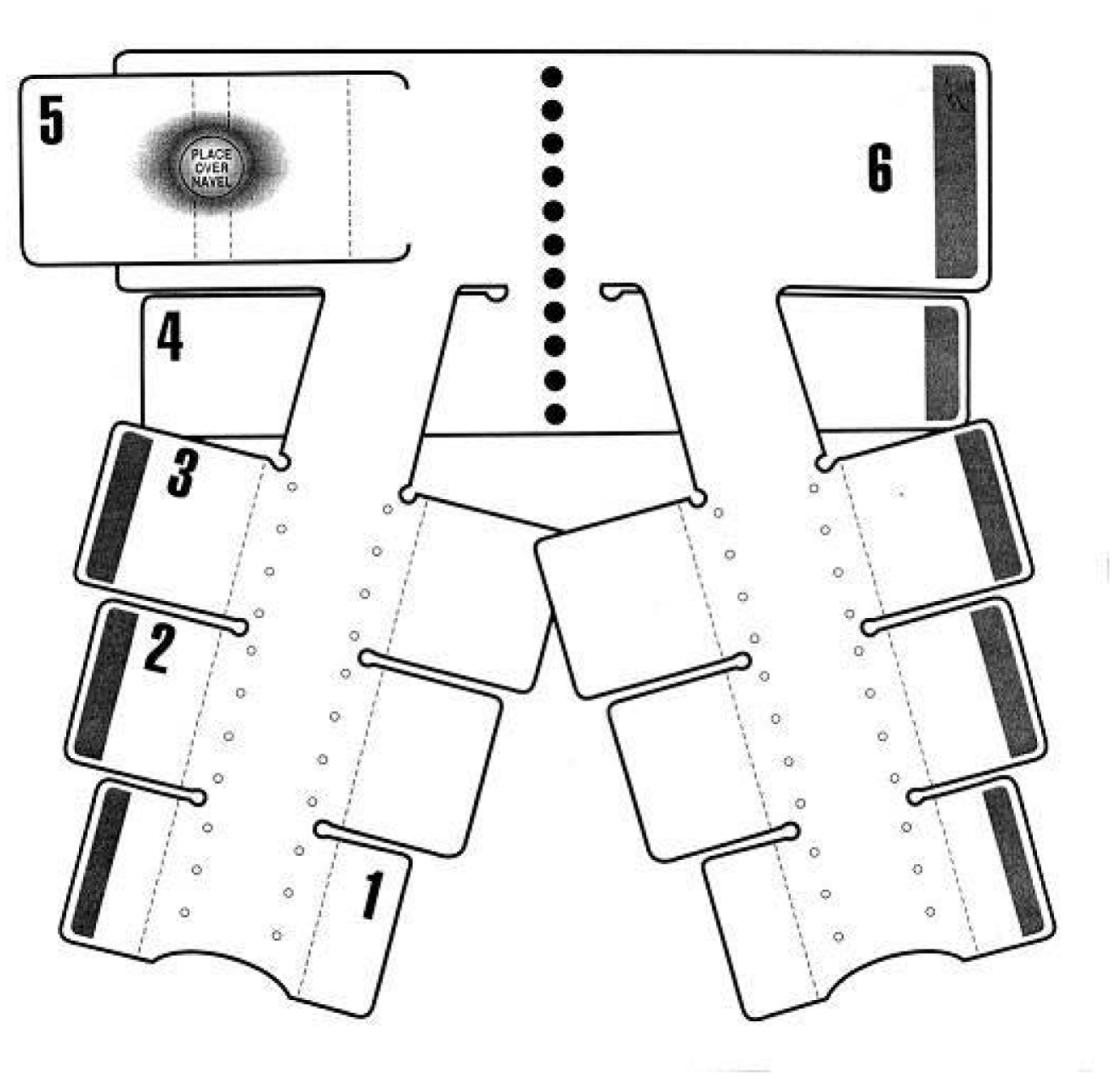
Schematic of the NASG. This figure shows an opened NASG. The articulated leg segments (1, 2, 3) are attached to the pelvic segment (4), and the abdominal segments (5, 6) contain a foam ball for extra pressure.

The Non-pneumatic Anti-Shock Garment (NASG) is a first-aid device that may assist women to survive delays in transport and therefore receive definitive treatment (Figure 1). The NASG, made of neoprene and Velcro™, compresses the lower body with nine articulated segments closed tightly around the legs, pelvis, and abdomen. A foam ball in the abdominal segment increases compression (Figure 2). Circumferential compression reduces vascular volume under the compressed areas, while expanding the central circulation by increasing preload, peripheral resistance, and cardiac output. Tamponade of abdominal, pelvic, and uterine vessels reduces blood loss [8]–[10].

The NASG is ideal for OH for a number of reasons. The abdominal panel stretches so that external uterine massage or compression can be accomplished. The design permits perineal access for performing vaginal procedures (suturing lacerations, manual exploration of the uterus and/or bimanual compression) or for inserting urinary catheters. Surgery can be performed by simply opening the abdominal segment immediately prior to beginning surgery, and then replacing this segment when surgery is completed; removal of the device for surgery is not necessary. The majority of the pressure exerted by the device is in the abdomen, retroperitoneum, and pelvis, reducing blood flow in OH immediately upon application [8], [10]–[13]. The NASG is very simple to apply and training in application is rapid.

The NASG was developed in the 1970s by the United States (US) National Aeronautics and Space Administration (NASA)/Ames; the original patent has expired. Although it has been used in the US for pre-hospital lower body trauma, the device was not used for obstetric hemorrhage in limited-resource settings until 2002 [14]. Unlike the predecessor device, the pneumatic anti-shock garment (PASG; also known as medical/military anti-shock trousers, MAST), the NASG has no air bladders, tubing, or gauges, so it is much simpler to use and cannot be over-inflated [15]. The PASG/MAST had a history of some adverse effects that might be related to over-inflation [16], [17]. The lower pressure exerted by the NASG may not increase risks of negative effects (dyspnea, decreased urine output, compartment syndrome, or ischemia to compressed areas). Furthermore, the PASG has been cited in a Cochrane Review as being either ineffective or perhaps harmful for the pre-hospital treatment of trauma patients, based on randomized trials, although the quality of those trials was criticized [18].

Two quasi-experimental studies with pre-intervention phases followed by NASG intervention phases demonstrated significantly reduced measured blood loss [19]–[22], faster shock recovery [23], and decreased mortality (RR 0.56, 95% CI 0.35–0.89) [19] or extreme adverse outcomes (severe morbidity and mortality combined) (RR 0.32, 95% CI 0.16–0.63) [20] with the use of NASGs for women with severe OH (>1000 mL) and shock at tertiary facilities [19]–[23]. A recent cost effectiveness analysis of that data at the tertiary level shows NASGs to be very cost effective for severe shock [24]. A systematic review of these quasi-experiments resulted in a World Health Organization (WHO) recommendation that the NASG be used as a temporizing measure while awaiting transfer [2], but noted that research evaluating potential benefits, potential harm and use at the primary level was needed [25].

This study was conducted to evaluate whether NASG application before transport from midwife-staffed primary health care centers (PHCs) would result in reduced maternal mortality for women with hypovolemic shock secondary to OH, reduced time to shock recovery, and if NASG use increased negative side effects. Zambia, with a maternal mortality ratio of 591 per 100,000 live births [26], and Zimbabwe, with a maternal mortality ratio of 960 per 100,000 live births, were the settings for the study [27].

## Methods

### Ethics Statement

Institutional review boards affiliated with the following institutions reviewed and approved study protocols and the informed consent document and process: University of California, San Francisco (UCSF); University of Zambia, Lusaka; University of Zimbabwe-UCSF Collaborative Programme on Health Research; and the Department of Reproductive Health and Research of the World Health Organization. Written informed consent (signature from literate participants and thumb print from those who could not sign) was obtained from all study participants who were conscious and able to give consent. All ethics committees approved a waiver of consent for unconscious women; written consent for an unconscious woman was either obtained from a relative and/or the patient after she regained consciousness. No data were entered for data collection forms that were not accompanied by a signed consent form. Figure 1, a photograph of a model in an NASG, was not taken during the study, but is a similar image used for illustrative purposes only. The subject of the photograph has given written informed consent, as outlined in the PLOS consent form, to publication of her photograph.

### Study Design

To test the efficacy and safety of the NASG, a cluster randomized control trial (CRCT) with PHCs as the cluster units was conducted in 38 PHCs referring to one of five Referral Hospitals (RHs) in Harare, Zimbabwe, and Lusaka and the Copperbelt Province, Zambia. (The protocol for this trial and supporting CONSORT checklist are available as supporting information; see Checklist S1 and Protocol S1.) We chose a cluster randomized design because it is impossible to blind providers to the intervention or to develop a placebo garment. Furthermore, it is difficult to require providers to randomize individual patients once they have had the opportunity to use the NASG and see the apparent results (decreased bleeding and improved vital signs).

The University of California, San Francisco (UCSF), University of Zambia, Lusaka, and University of Zimbabwe-UCSF Collaborative Programme on Health Research, Harare, conducted the study. The Department of Reproductive Health and Research of the UNDP/UNFPA/UNICEF/WHO/World Bank Special Programme of Research, Development and Research Training in Human Reproduction (HRP) served as the Data Coordinating Center (DCC). The Institute for Clinical Effectiveness and Health Policy (IECS), Buenos Aires, Argentina, conducted data monitoring and analysis. All institutional review boards/ethical committees approved the protocols.

Because the sites were research naïve, lacked baseline data, and clinicians were unfamiliar with NASGs, the study was implemented in three phases. Although the timelines were slightly different, all sites conducted each phase. The first phase, 2007–2008, was an 11-month preparatory phase, to familiarize the clinicians/data collectors with accurate form completion and adherence to standardized protocols. In the second phase, 10 months in 2008–2009, we implemented the NASG at the RHs, to give clinicians experience with it and to collect baseline outcome data. In the final phase, 2009–2012, the PHCs were randomized and NASGs implemented in the intervention group.

Based on baseline data, a covariate-constrained randomization procedure was used to ensure that intervention and control PHCs were balanced on number of deliveries, number of deliveries per midwife, distance to the RH, and proportion of OH cases expected [28]. The DCC allocated the PHCs to intervention or control group; allocation assignment was known by PHC staff and health authorities, but not by women in the PHC catchment communities. Because the NASG is visible, blinding of participants and clinicians/data collectors was impossible; the UCSF research team was blind to outcomes.

### Participants

#### Clusters

Eligible PHCs were peri-urban, had at least 500 annual deliveries conducted by midwives, and referred OH cases (≥500 mL blood loss by visual estimation) to one of five study RHs. Lusaka and Harare began in 2007; the Copperbelt Province was added in 2008. PHCs each covered the public assistance population in a given geographic catchment area. All PHCs had a maternity department, where midwives attended deliveries; any woman >24 weeks gestation with bleeding would be seen in the maternity department. Midwives were trained to provide prophylactic uterotonics, treat PPH with uterotonics and IV fluids, repair first- and second-degree perineal lacerations, and refer any patient with estimated blood loss ≥500 mL to the RH. Women with early pregnancy bleeding of <24 weeks gestation (ectopic pregnancy, complications of abortion, trophoblastic/molar pregnancy) were seen in the outpatient department by either midwives or clinical officers; these women in early pregnancy were also referred to the RH for bleeding ≥500 mL. No PHC was equipped to provide blood transfusion, surgery, or manual vacuum aspiration (MVA). All PHCs had access to a shared ambulance dispatch system to request ambulance transfer for their patients to the designated RH.

#### Individual participants

Participants were included in a cluster if they sought maternity care in a study PHC in their neighborhood. Participants were women with OH from any etiology and hypovolemic shock, with at least two of the following eligibility criteria: visually estimated blood loss ≥500 mL, pulse ≥100 BPM, systolic blood pressure ≤100 mm Hg. Women with antepartum hemorrhage were excluded if the fetus was viable. Participants were consented when they became eligible, if they were conscious and able to give consent. All ethics committees approved waiver of consent for unconscious women; consent for an unconscious women was either obtained from a relative and/or the patient after she regained consciousness.

### Interventions

#### PHC level interventions

The main intervention was applying the NASG first-aid compression device (Zoex, Colma, CA, USA). The NASG was rapidly applied, sequentially starting at the ankles, as the first step in shock resuscitation, and an absorbent perineal pad (Stay Dry Briefs, McKesson, San Francisco, CA, USA) was applied for blood loss measurement. Control group patients also had absorbent perineal pads applied at study entry. Both groups received the same hemorrhage/hypovolemic shock protocol: intravenous (IV) fluids, uterotonics and uterine massage (for uterine atony), and suturing of first- and second-degree lacerations.

#### Management at the RH

Women in the control arm also received the NASG upon RH arrival; those in the intervention arm remained in the NASG. The rationale for giving all women in the study the NASG at the RH was a) by the start of the RCT in 2009, the RHs were using the NASG on OH patients in the facility, b) the providers would find it difficult to refrain from applying the NASG after seeing the benefits at the RH level, and c) the goal of the study was to determine the efficacy when applied earlier in the OH/shock trajectory.

All women received standard shock/hemorrhage protocol: oxygen, IV fluids, uterotonics/uterine massage (for uterine atony), suturing of lacerations, manual removal of placenta or retained tissues, MVA, surgery, and blood transfusion, as necessary. The only differences in treatment received depended on hemorrhage etiologies, e.g. ruptured ectopic pregnancies required surgery. Upon RH arrival, all women had absorbent pads removed and weighed to determine blood loss during transport and had a calibrated blood measurement drape (Brass V Drape, Excellent Fixable Drapes, Madurai, India) placed. Staff at the RH removed the NASG when both criteria were met: the patient’s bleeding decreased to <50 mL per hour and the pulse was <100 BPM for two hours. Removal was incremental, beginning at the NASG ankle segments; vital signs were monitored for fifteen minutes to ensure hemodynamic stability before proceeding to subsequent segments.

### Data Collected

Data collected included reason for patient admission, age, gravidity, parity, weeks gestation, delivery information, prophylactic uterotonics, time hemorrhage started, treatment uterotonics, IV fluids, blood transfusions, and hemostatic procedures and/or surgeries. Blood loss in the drape and urine output in urine collection bags were recorded hourly, and vital signs were recorded every 15 minutes from study entry to NASG removal. Mean Arterial Pressure (MAP) <60 mmHg, ([(2 × diastolic)+systolic]/3), defined more serious shock [29]. Mortality, morbidities, diagnosis, and negative side effects that could potentially be attributed to the NASG (respiratory distress, reduced urine output, nausea, vomiting, abdominal pain) were recorded. In-facility clinicians/data collectors or study-funded midwives (when available) recorded data.

### Data Management

Data forms were reviewed by study coordinators and checked against medical records to resolve inconsistencies. Annually, a random sample of 10% of data forms was checked against medical records to confirm accuracy. Data were double-entered in OpenClinica (Akaza Research, Waltham, MA, USA), queried and cleaned, and analyzed using SAS (SAS Institute, Cary, NC, USA) and STATA (STATA Corp, College Station, TX, USA).

### Outcomes

The primary outcomes of the trial were the frequency of maternal mortality; survival with severe maternal morbidity; and extreme adverse outcome (EAO), a composite outcome of the two. Mortality was defined as dying before hospital discharge, as women were not followed up after discharge. Severe morbidity was defined as end-organ failure (cardiac, pulmonary, renal, cerebral) persisting 24 hours or more beyond shock resuscitation [30]. Patients who were lost to follow-up between the PHC and the RH were tracked to determine if there were outcome data on mortality. (See Text S1 for a detailed description of the lost to follow-up protocol).

Secondary outcomes included median blood loss measured by weighing the absorbent pad(s) upon RH admission; blood loss measured in the drape at the RH; blood loss during surgery; frequency of emergency hysterectomy for intractable uterine atony; and time to recovery from shock, defined as return to Shock Index (SI) <0.98 (SI = Heart Rate/Systolic Blood Pressure) [31]. Negative effects that might be attributable to the NASG included decreased urine output, respiratory difficulties, nausea, vomiting, and abdominal pain.

Co-interventions included: IV fluids, blood transfusions, receipt of the NASG at the RH, and duration of NASG use.

### Statistical Methods

Sample size was estimated in Acluster (Metaxis, Inc., Vista, CA) using the incidence rate of the primary composite EAO, based on an NASG pilot study conducted in Nigeria in 2005 (9% incidence, 50% effect size reduction) [32]. A sample size of 2400 women was calculated based on a reduction in incidence of EAO from 9.0% to 4.5% in EAOs at 20 clinics, of varying sizes, 80% power, two-sided type 1 error rate of 5%, and an intra-cluster correlation coefficient of 0.01 [33]. Achieving this target required enrolling approximately 3.3 women per clinic per month over 3 years, which we felt was possible based on an assessment of delivery rates and reported OH rates in Harare and Lusaka conducted while writing the proposal.

Initially, 12 clusters in Lusaka and 12 clusters in Harare were included with a planned enrollment period of three years. During the baseline period, an additional 14 clusters in the Copperbelt Province, Zambia were added in 2008 because the accrual rate was lower than expected, mainly due to lower than expected incidence of OH/shock and lower incidence of EAOs (5%). In 2008, prior to randomization, the Data Safety and Monitoring Board (DSMB) reviewed the enrollment and outcome rates from the baseline data collection phase and they changed the sample size target to 1944 women.

The DSMB performed power calculations in April 2011 and February 2012. In both instances the DSMB noted that accrual was low, but recommended continuing the study and either increasing clusters or extending enrollment. However we were unable to secure additional funding; enrollment ended May 2012, approximately 30 months post-randomization.

We undertook intention-to-treat analyses to compare treatment groups with a pretest-posttest design, where the outcome rates were measured at the cluster level prior to random assignment and again after randomization and implementation of intervention. As pre-specified in the protocol, two sets of analyses were conducted, both accounting for the cluster randomized study design: a) post-test observations only and b) post-test observations adjusted for baseline measurements. We estimated random effects logistic regression models for binary outcomes. To adjust for the outcome measurements at baseline, we included the logit of the cluster specific outcome rate as a covariate [34]. Diagnosis was entered in the regression model to adjust for an imbalance among participants recruited after randomization. The effect size was reported as OR with 95% CIs. For continuous outcomes, a random effects linear regression model was estimated. Measured blood loss values were transformed into the log metric for normality, and the ratio of the geometric mean and its 95% CI were reported. To compare SI recovery trajectories, Cox regression models were estimated to evaluate group differences accounting for study design effects by including a working correlation matrix to adjust the standard errors. Statistical tests were 2-sided and performed at the 5% significance level.

This study is registered with ClinicalTrials.gov number, NCT00488462.

## Results

**Figure 3:**
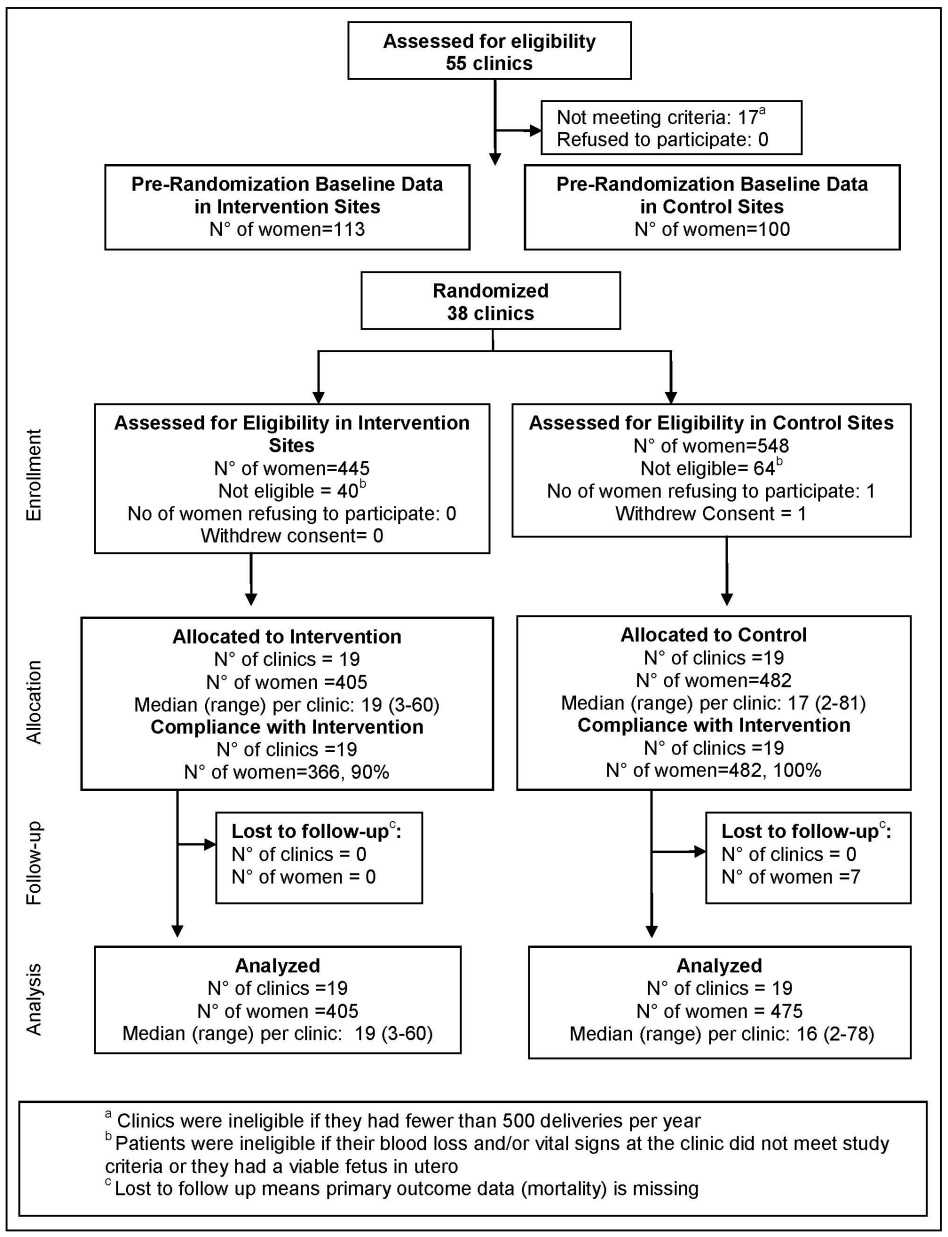
Cluster Randomized Trial Design.

Figure 3 shows the trial profile. Fifty-five clinics were assessed for eligibility; thirty-eight met criteria and were included in the baseline period and subsequently randomized. During the baseline period, 114 women and 99 women were enrolled in what would become, after the clinics were randomized, the intervention and control groups respectively. After randomization, 548 women at the control PHCs were assessed for eligibility and 482 were allocated to the control group; 445 women at the intervention PHCs were assessed for eligibility and 405 were allocated to the intervention group. No clusters were lost to follow-up and all clusters enrolled participants. Seven women were lost to follow-up in the control group; none in the intervention group. (See Text S1 for a detailed description of the lost to follow-up protocol.) Among women in the intervention group, 366 (90%) received the NASG at the PHC; none received it at the PHC in the control group.

### PHC and Participant Characteristics

As shown in Table 1, PHC characteristics measured at baseline were comparable between groups. Among women recruited during the baseline period, mortality was higher in the clinics later randomized to the intervention group. Other baseline variables were similar, including diagnoses. Women in both groups enrolled during the intervention period (after randomization) were similar, with the exception of diagnosis: abortion and placental abruption were more common in the control group; uterine atony, retained placenta, placenta accreta, and lacerations were more common in the intervention group (Table 2). Women in the intervention group spent a median of 100 minutes (IQR 70–135) in the NASG between study enrollment and arrival at the RH, women in the control group spent a median of 110 minutes (IQR 78–155) between study enrollment and being placed in the NASG at the RH (not shown). Median transfer time was 40 minutes for the intervention group (IQR 30–60) and 49 minutes for the control group (IQR 33–67) (not shown).

**Table 1:** Clinic and Women’s Characteristics and Outcome Assessment during Baseline Period.

				Intervention Group	Control Group
				(Number of clinics = 19)	(Number of clinics = 19)
				n (%)	n (%)
**Clinic characteristics at baseline** a	Volume of births		Low (<1235 births/yr)	5 (26%)	5 (26%)
			Medium (1235–2751 births/yr)	9 (48%)	10 (53%)
			High (>2751 births/yr)	5 (26%)	4 (21%)
	N° of midwives		Low (<8)	2 (11%)	4 (21%)
			Medium (8–15)	13 (68%)	11 (58%)
			High (>15)	4 (21%)	4 (21%)
	Distance to RH (km)^b^			12.19 (5.22)	11.84 (6.25)
**Characteristics of women at baseline**	N° of women			114	99
	Ageb,c			26.8 (5.4)	26.6 (5.7)
	Parityd,e			2 (1–3)	2 (1–4)
	Gestational ageb,f			36.8 (3.0)	37.1 (2.4)
	Diagnosis	Complications of Abortion		15/113 (13.3%)	18/99 (18.2%)
		Postpartum Uterine Atony		25/113 (22.1%)	31/99 (31.3%)
		Retained Placenta		33/113 (29.2%)	28/99 (28.3%)
		Lacerations/Genital Trauma		22/113 (19.5%)	16/99 (16.2%)
		Placental Abruption		10/113 (8.9%)	4/99 (4.0%)
		Placenta Previa		4/113 (3.5%)	0/99 (0.0%)
		Ruptured Uterus		3/113 (2.7%)	0/99 (0.0%)
		Ectopic Pregnancy		0/113 (0.0%)	1/99 (1.0%)
		Placenta Accreta		1/113 (0.9%)	0/99 (0.0%)
		Molar Pregnancy		0/113 (0.0%)	0/99 (0.0%)
		Other		0/113 (0.0%)	1/99 (1.0%)
	Estimated REVEALED blood loss at study entry (mL)^d^			600 (450–800)	500 (350–600)
	MAP<60 at study entry^g^			20/114 (17.5%)	25/96 (26.0%)
	Unconscious at study entry			1/114 (0.9%)	0/99 (0.0%)
**Outcome assessment at baseline**	Survived with severe morbidity^h^			0/114 (0.0%)	1/99 (1.0%)
	Mortality			6/114 (5.3%)	3/99 (3.0%)
	Extreme adverse outcome			6/114 (5.3%)	4/99 (4.0%)

a Variables used for randomization, using Phase 1 clinic statistics.

b Mean (Standard Deviation).

c N° of women in the Intervention Group: 113; N° of women in the Control Group: 99.

d Median (Interquartile Range) is reported.

e N° of women in the Intervention Group: 104; N° of women in the Control Group: 90.

f For those ≥24 weeks; does not include cases with molar or ectopic pregnancies or abortion. N° of women in the Intervention Group: 92; N° of women in the Control Group: 69.

g MAP was measured as ([(2 * diastolic BP)+systolic BP]/3); includes women with non-palpable BP.

h Includes acute renal failure, acute respiratory distress syndrome, heart failure, cerebral impairment (seizures, unconsciousness, motor/cognitive loss) among women who survived.

**Table 2:** Women’s Characteristics Enrolled during Intervention Period.

			Intervention Group	Control Group
			n (%)	n (%)
**Characteristics of women enrolled during intervention period**	N° of women		405	482
	Agea,b		26.9 (5.9)	27.3 (6.3)
	Parityc,d		2 (1–3)	2 (1–3)
	Gestational agea,e		37.7 (2.6)	37.4 (2.9)
	Diagnosis^f^	Complications of Abortion^k^	73/405 (18.0%)	177/478 (37.0%)
		Postpartum Uterine Atony^j^	163/405 (40.3%)	146/478 (30.5%)
		Retained Placenta^i^	97/405 (24.0%)	85/478 (17.8%)
		Lacerations/Genital Trauma^j^	51/405 (12.6%)	34/478 (7.1%)
		Placental Abruption^i^	7/405 (1.7%)	19/478 (4.0%)
		Placenta Previa	3/405 (0.7%)	5/478 (1.1%)
		Ectopic Pregnancy	3/405 (0.7%)	5/478 (1.1%)
		Ruptured Uterus	2/405 (0.5%)	5/478 (1.1%)
		Placenta Accreta^i^	5/405 (1.2%)	0/478 (0.0%)
		Molar Pregnancy	1/405 (0.3%)	2/478 (0.4%)
	Estimated REVEALED blood loss at study entry (mL)c,g		500 (480–700)	500 (500–800)
	MAP<60 at study entry^h^		129/399 (32.3%)	149/475 (31.4%)
	Unconscious at study entry		11/403 (2.7%)	13/477 (2.7%)

Note: Wilcoxon Rank Sum test utilized to test all continuous variables due to non-normality. Chi-square test used for categorical values except where noted.

a Mean (Standard Deviation).

b N° of women in the Intervention Group: 404; N° of women in the Control Group: 476.

c Median (Interquartile Range) is reported.

d N° of women in the Intervention Group: 404; N° of women in the Control Group: 472.

e For those ≥24 weeks; does not include cases with molar or ectopic pregnancies or abortion. N° of women in the Intervention Group: 291; N° of women in the Control Group: 250.

f Fisher’s exact test used for categorical values.

g N° of women in the Intervention Group: 391; N° of women in the Control Group: 447.

h MAP was measured as ([(2 * diastolic BP)+systolic BP]/3); includes women with non-palpable BP.

i Significant difference between intervention groups where p<0.05.

j Significant difference between intervention groups where p<0.01.

k Significant difference between intervention groups where p<0.001.

### Co-Interventions

Results on co-interventions: IV fluids, blood transfusions, and receipt of NASG at RH (Table 3), show a significant difference only in rapidity of receipt of blood transfusions, with more women in the NASG receiving blood within one hour of arrival at RH (OR 3.21, 95% CI 1.23–8.35, p = 0.02). However, the overall rate of transfusions between groups was similar; 42% intervention vs. 38.6% control (OR 1.34, 95% CI 0.94–1.92, p = 0.11). Of the women in the control group, 13.2% did not receive the NASG at the RH and 18.8% of women in the intervention group who had not received the NASG at the PHC also did not receive it at the RH (not shown). The median number of minutes in the NASG at the RH was 375 for women in the intervention group compared to 420 for women in the control group (OR 0.91, 95% CI 0.77–1.08, p = 0.28).

**Table 3:** Co-interventions at the Referral Hospital.

	Intervention Group	Control Group	Odds Ratio	P-value
	(n = 405)	(n = 475)	(95% CI)	
	n/N (%)	n/N (%)		
Minutes in NASG at RHa,b	375 (240–588)	420 (280–683)	0.91 (0.77–1.08)^c^	0.28
Women receiving >1500 mL of IV fluids within 1 hour of study admission^d^	59/402 (14.6%)	57/456 (12.5%)	1.20 (0.78–1.87)	0.41
Women with uterine atony who received uterotonics within1 hour of study admission	84/161 (52.2%)	68/138 (49.3%)	1.32 (0.60–2.86)	0.49
Women receiving blood transfusion within1 hour of hospital admission	32/398 (8.0%)	19/435 (4.4%)	3.21 (1.23–8.35)	0.02
Women receiving blood transfusion within 2 hours of hospital admission	88/398 (22.1%)	73/435 (16.8%)	1.98 (1.02–3.86)	0.04
Women receiving blood transfusion ever	167/398 (42.0%)	168/435 (38.6%)	1.34 (0.94–1.92)	0.11
Time to blood transfusion from RH arrivala,e	117.5 (75–265)	135 (90–270)	0.78 (0.52–1.17)^c^	0.23
Time to blood transfusion from study entrya,f	229 (165–380)	260 (195–420)	0.93 (0.67–1.29)^c^	0.65

a For each group the median and the interquartile range is reported.

b N° women in the Intervention Group: 381; N° women in the Control Group: 268.

c For the estimation of the effect the variable was transformed into the log metric for normality and the ratio of the mean is reported.

d The protocol asked for 1500 mL to be administered in the first hour of resuscitation.

e N° women in the Intervention Group: 166; N° women in the Control Group: 163.

f N° women in the Intervention Group: 166; N° women in the Control Group: 162.

### Outcomes

Outcomes for the intention-to-treat analysis are in Table 4. The intervention was associated with a 46% reduction in the odds of mortality (OR 0.54, 95% CI 0.14–2.05, p = 0.37) and 54% reduction in the odds of the composite EAO (OR 0.46, 95% CI 0.13–1.62, p = 0.22); these differences were not statistically significant. The results were similar after adjusting for the outcome rates measured during the baseline period (mortality: AOR 0.55, 95% CI 0.14–2.18, p = 0.40; EAO: AOR 0.46 95% CI 0.13–1.67, p = 0.24), and after adjusting for both the rate of outcomes at baseline and definitive diagnosis imbalance, mortality: AOR 0.47 (95% CI 0.12–1.87, p = 0.28) and EAO: AOR 0.39 (95% CI 0.11–1.44, p = 0.16) (not shown).

**Table 4:** Primary and Secondary Outcomes.

		Intervention Group	Control Group	ICC	Odds Ratio	P-value
		(n = 405)	(n = 475)		(95% CI)	
		n/N(%)	n/N(%)			
**Primary Outcomes**	Survived with severe morbidity^a^	0/403 (0.0%)	1/465 (0.2%)		–	–
	Mortality	4/405 (1.0%)	11/475 (2.3%)	0.022	0.54 (0.14–2.05)	0.37
	Extreme adverse outcome	4/403 (1.0%)	12/465 (2.6%)	0.019	0.46 (0.13–1.62)	0.22
**Secondary Outcomes**	Blood loss in transit (mL)b,c	205 (105–405)	218 (95–461)		1.04 (0.80–1.36)^d^	0.75
	Blood loss after arrivalc,e	60 (30–280)	50 (30–150)		1.31 (0.79–2.16)^d^	0.30
	Total blood lossc,f	355 (160–655)	336 (145–599)		1.06 (0.76–1.49)^d^	0.73
	Emergency hysterectomy^g^	1/240 (0.4%)	0/296 (0.0%)		–	–
	Minutes to normal Shock Index^h^	170 (96–299)	209 (114–386)		1.25 (1.02–1.52)^i^	0.03

a Includes acute renal failure, acute respiratory distress syndrome, heart failure, cerebral impairment (seizures, unconsciousness, motor/cognitive loss) among women who survived.

b The blood loss in transit was measured in 155 women in the Intervention Group and 175 women in the Control Group.

c For each group the median and the interquartile range is reported.

d For estimation of the effect the variable was transformed into the log metric for normality and the ratio of the mean is reported.

e Blood loss after arrival was measured in 267 women in the Intervention Group and 269 women in the Control Group at post-randomization.

f Total blood loss was measured in 125 women in the Intervention Group and 123 women in the Control Group at post-randomization.

g Hysterectomy among women with diagnosis of uterine atony and complications of abortion.

h Shock Index was calculated by (heart rate/systolic blood pressure). For each group the median and interquartile range and hazard ratio is reported. Shock Index was measured in 326 women in the Intervention Group and 358 women in the Control Group at post-randomization.

i Hazard ratio.

Only one secondary outcome was statistically significant. The median time in minutes to return to SI <0.98 (HR 99/SBP101) was 170 minutes (IQR 96–299) for the intervention group vs. 209 minutes (IQR 114–386) for the control group (HR 1.25 (95% CI 1.02–1.52, p = 0.03), indicating a 25% faster rate of recovery for women in the intervention group.

There were no significant differences by group for women who experienced negative side effects at the PHC. At the RH, women in the intervention group were more likely to have reported abdominal pain (OR 1.96, 95% CI 1.12–3.45, p = 0.02) (Table 5). No women required removal of the NASG because of pain.

**Table 5:** Side Effects.

		Intervention Group	Control Group	Odds Ratio	P-value
		(n = 405)	(n = 475)	(95% CI)	
**At the clinic**	Respiratory symptoms/dyspnea	33/382 (8.7%)	23/420 (5.5%)	1.68 (0.88–3.22)	0.12
	Abdominal pain	190/383 (49.6%)	243/426 (57.0%)	0.77 (0.50–1.19)	0.24
	Nausea	26/383 (6.8%)	35/421 (8.3%)	0.93 (0.45–1.94)	0.85
	Vomiting	15/382 (3.9%)	27/422 (6.4%)	0.66 (0.31–1.38)	0.27
**At the RH**	Respiratory symptoms/dyspnea	12/394 (3.1%)	19/437 (4.4%)	0.90 (0.34–2.36)	0.83
	Reduced urine output	2/394 (0.5%)	3/438 (0.7%)	0.74 (0.12–4.46)	0.74
	Abdominal pain	213/395 (53.9%)	179/437 (41.0%)	1.96 (1.12–3.45)	0.02
	Nausea	9/393 (2.3%)	7/437 (1.6%)	1.45 (0.50–4.26)	0.49
	Vomiting	11/393 (2.8%)	6/437 (1.4%)	2.13 (0.76–5.97)	0.15

## Discussion

Women with hypovolemic shock secondary to OH at the PHC transported to an RH in the NASG had a non-significant unadjusted 46% lower mortality, and 54% lower composite EAO, than women in the control group, with an adjusted 61% decrease in EAO. Women in the intervention group also had a 25% statistically significant reduction in time to lower Shock Index. These results suggest a treatment benefit from earlier application of the NASG. As expected, other secondary outcomes were not different, as all women received the NASG at the RH. There were no significant negative effects from NASG use at the PHCs, but an increase in abdominal pain was reported at the RH.

The difference in median length of time in the NASG between groups was only 55 minutes. The median time in NASG for women in the intervention group was 100 minutes before RH arrival and 375 minutes after arrival (475 total), while the women in the control group were in the NASG at the RH for 420 minutes. These results suggest that it is not the length of time, but the earlier application that affected the outcomes. We also interpret the significantly more rapid recovery of the SI to <0.98 to be a result of earlier application of the NASG. An alternative explanation could be the more rapid administration of blood transfusions, as a higher proportion of the intervention group received a blood transfusion in the first two hours after RH admission. However, there was no difference between the two groups in the median time from study entry or RH entry to time of blood transfusion (Table 3). It is more likely that the observed effect was due to the earlier application of the NASG than to the blood transfusions. Further supporting evidence of this explanation is that median recovery time to SI in both groups was 50–60 minutes before the median time to blood transfusion. The median time to shock recovery in both the NASG group and the control group (170 minutes and 209 minutes, respectively) occurred before receipt of blood transfusions (229 minutes and 260 minutes, respectively).

Strengths of this study include that it was conducted in settings where the NASG was more likely to have a large impact. In limited-resource settings with high maternal mortality, PHCs and RHs are busy, understaffed, and often characterized by delays in transport and time to receipt of definitive therapies. Conducting a methodologically rigorous randomized trial under these conditions was a challenge, but doing so provides results that can be applied to similar settings. A further strength of this study was having mortality as an outcome. The reduction in maternal mortality observed in this trial is uniquely high for a single intervention. Measuring maternal mortality is difficult and it is rarely used as an outcome [35], [36]. A review of maternal health intervention studies showed only four with a mortality outcome [37]–[40]. Only one was a trial of a single medical intervention, the MAGPIE trial, a CRCT of magnesium sulphate for pre-eclampsia, which showed a non-significant 45% mortality reduction (RR 0.55, CI 0.26–1.14) [38]. Another strength of the trial was a high rate of follow-up on our primary outcome; <1% of individuals were missing data on mortality. However, missingness was higher for some secondary outcomes.

A major limitation to the study was low accrual. The lack of statistical significance on the primary outcome may be due to a smaller than expected number of women with OH at PHCs and a lower than expected event rate. (See Text S2 for more information on low accrual.) The low incidence of severe end-organ failure maternal morbidities (<0.2%) was not consistent with previous trials in which these rates were >3% [19], [21], mainly renal failure. A lack of nephrology units and dialysis may have contributed to a lack of survival with this morbidity. An alternative explanation could be that the training efforts made to assure protocol adherence improved patient outcomes, not an uncommon finding in trials conducted over time [41]. (See Table S1 for an explanation of study-sponsored trainings).

Although the sample size was not reached, our results are consistent with the hypothesized >50% reduction in EAOs and significantly more rapid decrease in SI among those treated with the NASG, with no increase in adverse health effects. These findings are similar to prior findings of the NASG at the tertiary level [19], [21], [23].

A finding that was not expected was a lack of a statistically significant difference in measured blood loss in transit (205 mL NASG vs. 218 mL control); measured blood loss has consistently been statistically significant in the quasi-experimental trials at the tertiary level [19]–[22]. However, 62% of data was missing on blood loss in transit, and 72% of women had no total blood loss recorded.

The two primary limitations to this study, lack of reaching sample size for power and high rate of missing values for blood loss in transit data, should be addressed. As we note in (Text S2), the rate of severe hemorrhage with hypovolemic shock was lower than we had anticipated. Given the increasing global practice of prophylactic uterotonics and improved management of early PPH, we feel that obtaining a sample size adequate to demonstrate a statistically significant decrease in maternal mortality, even with the large effect size of the NASG, in a cluster randomized trial, is unattainable. We do not feel that this type of randomized efficacy trial with detailed data collection will be able to be repeated in a period of time that would be supportable by the majority of funders. We therefore recommend more pragmatic trials with a rigorous evaluation component. The other primary limitation, the high rate of missing values for blood loss in transit, could be overcome in future studies by greater attention to weighing pads on arrival at the RH, and more careful calibration and use of electronic scales. While the amount of blood loss is a secondary outcome, it is also a proxy for the more crucial mortality outcome, and therefore understanding the effect of the NASG on blood loss during transit would still be of interest.

We did not expect to observe a statistically significant increase in abdominal pain at the RH. This was inconsistent with a prior quasi-experimental study that found no difference between study groups in experience of abdominal pain [42], [43]. While it can be unpleasant, abdominal pain is not a safety concern. The reason for the increase in reports of abdominal pain at the RH level in the intervention group is unclear. Perhaps the increase in this study might be related to different distribution of hemorrhage diagnosis, or a difference in surgeries and/or pain medications administered for post-surgical patients. Since the women in the intervention group actually had the NASGs on for less time at the RH (375 minutes vs. 420 minutes), we are unsure of the cause of the increased pain or what to do to alleviate it. This should be followed up in any future studies.

Another limitation was the imbalance in hemorrhage etiologies, which is difficult to explain given the balanced randomization and equal distribution of etiologies at baseline. The most significant differences appear to be the increased enrollment of postpartum hemorrhage (PPH) etiologies in the intervention group (uterine atony, retained placenta, genital lacerations, and placenta accreta). As noted in the methods and results sections, we conducted an adjusted analysis for the imbalance and found an even greater protective effect for the NASG. Inclusion criteria were the same at all PHCs and in all wards at the PHCs. However, the staff at intervention clinics had to do a new procedure, application of the NASG; this may have been more strictly adhered to in the maternity wards than in the outpatient departments where abortion patients were seen. It is also possible that the acuity of the PPH patients was perceived as more severe.

Despite these differences in etiologies, however, the most important prognostic factor, condition on study entry, was similar between study groups, with approximately 32% of women with MAP<60 mmHg [19], [44]. The overall effect of the imbalance in etiologies was recruitment of a population at higher risk in the intervention group, which might have negatively biased the comparison. However, adjustment for these etiologies post-randomization did not diminish the effect size. In fact the AOR for EAO was 0.39 (0.11–1.44, p = 0.16), actually strengthening our results, since the PPH etiologies are more likely to be associated with extreme adverse outcomes in this sample.

The slightly more rapid time between study enrollment and arrival at RH (100 minutes vs. 110 minutes) for the intervention group and the more rapid receipt of blood transfusions may reflect the NASG as a visual cue indicating severity and need for action. The low level of all women in the sample receiving blood transfusions (∼40%) does not necessarily reflect their condition; blood transfusions may be difficult to obtain in these settings, and the amount of blood transfused (or even ever receiving a transfusion) may be more a reflection of blood availability than patient need.

The generalizability of the findings is limited by the specific settings in Zambia and Zimbabwe and the nature of a research project compared to real-world clinical care. While the individual women are most likely similar to other women using public facilities in sub-Saharan Africa, the clusters may be different; most of the clinics were peri-urban, referred to teaching facilities, and staffed by midwives, nurses, or clinical officers. While the NASG is simple to apply, the training for the project also included frequent reviews of evidence-based protocols on prevention, identification, and management of OH/shock. Furthermore, there was supervision and protocol reinforcement associated with research.

Despite the lack of statistical significance, these findings on the primary outcome and the significantly faster shock recovery suggest that as a first-aid device, there might be a treatment benefit for NASG use at the PHC level, with no risk of safety issues. Concerns expressed by clinicians, based on previous experience with PASGs about safety (that the NASG might exert too much pressure, or increase the risk of anuria/oliguria or dyspnea), do not appear to be an issue. Currently there are no other tools available to stabilize women with hypovolemic shock and severe OH until definitive therapies can be reached and administered. Therefore, these results could be helpful in informing policy and clinical decisions to incorporate the NASG into health systems seeking to overcome delays contributing to maternal mortality.

Given the potential clinical benefit to application of NASG to women suffering hypovolemic shock, policy makers may be interested in investing in NASGs for their maternal health systems. Our understanding of the current treatment and prevention options for PPH indicate that a holistic approach to maternal mortality reduction would include: investments in misoprostol for prevention or treatment where safe oxytocin injection is not possible; referral and transport strengthening to enable community level, PHC, or BEmOC facilities to transport women in shock to CEmOC facilities; and the application of the NASG prior to transport. NASGs could also be placed on ambulances when hemorrhaging women are picked up for transport. If the NASG has not been applied prior to transport, then it could be applied at those CEmOC facilities that have delays. The NASG plays a unique role in hemorrhage and hypovolemic shock management. If a woman is given prophylaxis but still hemorrhages, or she has an etiology that is not responsive to uterotonics, the NASG might reverse shock more quickly and contribute to her surviving during transport or delays at referral centers. Therefore, systems should not consider investing in either uterotonics or the NASG, but in both: uterotonics to prevent and treat atonic PPH; and the NASG for shock reversal, more rapid recovery from shock, and for non-atonic hemorrhage etiologies.

The difficulty and expense of conducting another NASG RCT at the PHC level may preclude future efficacy trials. However, we believe that more research is needed; the next step could be a pragmatic multi-country study, set in high-volume facilities and referring communities with high maternal mortality, with a rigorous evaluation framework.

## Acknowledgments

We thank the many doctors, nurses, midwives, and health system staff in Lusaka District, Kalulushi District, Kitwe District, Ndola District, and University Teaching Hospital, Kitwe Central Hospital, and Ndola Central Hospital in Zambia; and the City Council Clinics and Pariyenatwa Group of Hospitals and Harare Central Hospitals in Harare, Zimbabwe, who collected and reviewed data and cared for women in this study. Field coordinators include Rhoda Amamfumba, Violet Mambo, Jessica DeMulder, Kathleen McDonald, Kelly Winter, Althea Anderson, Stephanie Boarden, and Ashley Leech. Many thanks as well to the students and interns who supported the project and did special sub-projects. We thank our Data and Safety Monitoring Board including Jose Belizan, Fernando Althabe, Shrikant Bangdiwala, Fridah Kazembe, and Anna Colletor-Penduka for their hard work and support. Also thanks to Daniel Giordano of the Centro Rosarino de Estudios Perinatales for initial database set up and to Sheri Lippman of UCSF for analysis and data cleaning oversight.

## Supporting Information

Table S1 **Study Sponsored Training Content and Schedule.** Study-sponsored trainings were conducted at the beginning of each of the three study phases. In addition, after randomization annual update/refresher trainings were held in each city (Harare, Lusaka, Kitwe or Ndola [Copperbelt cities] ) for all RH and PHC staff, and, as necessary, PHC-specific or unit-specific refresher trainings were held in places where enrollment was low or multiple errors in filling out data collection forms were reported. This table contains an outline of the various training program topics, their scheduling during the study phases, and their intended audience(s).
(PDF)

Text S1 **Lost to Follow-Up, Tracking Participants between PHCs and RHs.** Cases were considered lost to follow-up if there was no data on the primary outcome, mortality. Difficulties in communication between the PHCs and the RHs resulted in 7 of 887 total women (0.8%) having no primary outcome data available, and being considered lost to follow-up for the analysis, after the tracking methods described in this supplement were utilized.
(PDF)

Text S2 **Low Accrual.** Our estimated sample size was based on a pilot study conducted in Nigeria in 2004–2006 which found an adverse outcome rate of 9.0%. Based on a reduction from 9.0% to 4.5% in incidence of adverse outcomes, 80% power, two-sided type I error of 5%, and an intra-cluster correlation coefficient of 0.01, we calculated a sample size of 2400 women. By the time data collection began, mass forced migration of the peri-urban areas served by our study sites and uptake of pre-delivery uterotonic prophylaxis resulted in reductions in both the pool of delivering women and the actual incidence of PPH. Attempts to increase enrollment by adding 16 study sites in the Copperbelt region in 2008 did not result in increased clinic mean enrollment greater than 0.8 cases per clinic per month.
(PDF)

Checklist S1 **CONSORT Checklist.** The CONSORT 2010 checklist of information to include when reporting a cluster randomized trial.
(PDF)

NIH Protocol S1 **The NASG RCT protocol.**
(PDF)

Related Article S1 **A related article published in PLOS ONE.**
(PDF)
