# Supplementary material for: Non-Pneumatic Anti-Shock Garment (NASG), a First-Aid Device to Decrease Maternal Mortality from Obstetric Hemorrhage: A Cluster Randomized Trial
Source: PLoS One. 2013 Oct 23;8(10):e76477. doi: 10.1371/journal.pone.0076477 (PMC3806786; doi:10.1371/journal.pone.0076477)
Supplement: Table S1 — Study Sponsored Training Content and Schedule. Study-sponsored trainings were conducted at the beginning of each of the three study phases. In addition, after randomization annual update/refresher trainings were held in each city (Harare, Lusaka, Kitwe or Ndola [Copperbelt cities] ) for all RH and PHC staff, and, as necessary, PHC-specific or unit-specific refresher trainings were held in places where enrollment was low or multiple errors in filling out data collection forms were reported. This table contains an outline of the various training program topics, their scheduling during the study phases, and their intended audience(s). (PDF) [file pone.0076477.s001.pdf]

Table S1: Study Sponsored Training Content and Schedule

	**Baseline Phase 1** (No NASG)		**Baseline Phase 2** (NASG at RH only)	**CRCT Phase 3** (NASG at RH and Intervention clinics)	
**Training**	PHC staff	RH staff	RH staff	PHC Staff at Intervention Clinics	PHC Staff at Control Clinics*
Study Overview	X	X	X	X	X
Review of Standard Protocol for OH and Shock Management	X	X	X	X	X
Research Ethics and Informed Consent	X	X	X	X	X
How to Complete Study Forms	X	X	X	X	X
How to Use the NASG			X	X	

*At the conclusion of the study, How to Use the NASG training was conducted with clinic control staff.
