# Supplementary material for: Non-Pneumatic Anti-Shock Garment (NASG), a First-Aid Device to Decrease Maternal Mortality from Obstetric Hemorrhage: A Cluster Randomized Trial
Source: PLoS One. 2013 Oct 23;8(10):e76477. doi: 10.1371/journal.pone.0076477 (PMC3806786; doi:10.1371/journal.pone.0076477)
Supplement: Text S1 — Lost to Follow-Up, Tracking Participants between PHCs and RHs. Cases were considered lost to follow-up if there was no data on the primary outcome, mortality. Difficulties in communication between the PHCs and the RHs resulted in 7 of 887 total women (0.8%) having no primary outcome data available, and being considered lost to follow-up for the analysis, after the tracking methods described in this supplement were utilized. (PDF) [file pone.0076477.s002.pdf]

Text S1: Lost To Follow-Up, Tracking Participants Between PHCs and RHs

Cases were considered lost to follow-up if there was no data on the primary outcome, mortality. Because of difficulties in communication between the PHCs and the RHs, there were 46 of 887 (5.1%) women enrolled in the study at a PHC who did not have a study data form completed at the RH. Using the methods described below we tracked the 46 to find out the primary outcome data, and were able to find primary outcome data on all but 7 of 46 (15%) or 7 of 887 total (0.8%). Only these women could not be tracked as ever having arrived at the RH, and these were considered lost-to-follow up for the analysis.

All patients who were confirmed as eligible enrolled cases without a completed study form at the RH had their outcomes documented on a Lost to Follow-Up form. These forms contained information on patient eligibility, referral to the hospital, and outcomes at the hospital. To obtain this information we checked clinic records to verify that there was a documented case of OH that was sent to the RH on the date in question. We then verified with hospital admissions books that the patient arrived and was admitted. Once arrival at the RH was verified, we checked the medical record, if available. If the medical record was unavailable, we counterchecked the facility roster of all maternal deaths for a patient of the same name, age, definitive diagnosis, from the same clinic on the same date or within one month.

A total of 46 women out of 887 (5.2%) were enrolled but had incomplete data. Of these, more than half of the women (27/46, 58%) had clearly documented outcomes in medical records. Another quarter (12/46, 26%) were verified as having arrived and had no matching record in the maternal death records. Only 7 of 46 (15%) or 7 of 887 total (0.7%) could not clearly be traced as ever having arrived at the RH and were considered lost to follow-up in the analysis, since we have no data on the primary outcome of mortality.

It is possible these patients refused the hospital referral and went home before the ambulance arrived; that they were not actually in hypovolemic shock and stabilized at the clinic after receiving IV fluids and were discharged from the clinic, therefore not ever actually referred to the RH; that the ambulance did not come and they arranged their own transport and went home rather than to the hospital; or that they went to the hospital but were not registered in the admissions book. Without this information, we had no way to trace their medical record. However, it is unlikely that any of these seven cases died. If they had died en route in an ambulance or private conveyance they would have been registered in the admissions books at the hospital. If they had gone home and died, the family would still have brought the body to the hospital to obtain a death certificate and this information would have been noted in the hospital admissions book. There is a remote possibility that the patient could have died and the family took her body to the hospital Casualty Department instead of the Obstetrics and Gynecology Department, but Casualty is more crowded and includes accidents and all other deaths so families will generally bring deceased mothers to Obstetrics and Gynecology, especially if there had been a live birth as they would also have had to register the baby at Obstetrics and Gynecology (Rhoda Amafumba in conversation with Elizabeth Butrick December 13, 2012).

Control patients were more likely than intervention patients to be lost to follow-up because intervention patients arrived at the RH already wearing the NASG – a much more obvious sign that they were enrolled in the study. Control patients comprised 40 of the 46 (87%) of the patients who had their outcomes tracked using Lost-to Follow Up forms and all 7 of the women with unknown outcomes.
