# Supplementary material for: Non-Pneumatic Anti-Shock Garment (NASG), a First-Aid Device to Decrease Maternal Mortality from Obstetric Hemorrhage: A Cluster Randomized Trial
Source: PLoS One. 2013 Oct 23;8(10):e76477. doi: 10.1371/journal.pone.0076477 (PMC3806786; doi:10.1371/journal.pone.0076477)
Supplement: Text S2 — Low Accrual. Our estimated sample size was based on a pilot study conducted in Nigeria in 2004–2006 which found an adverse outcome rate of 9.0%. Based on a reduction from 9.0% to 4.5% in incidence of adverse outcomes, 80% power, two-sided type I error of 5%, and an intra-cluster correlation coefficient of 0.01, we calculated a sample size of 2400 women. By the time data collection began, mass forced migration of the peri-urban areas served by our study sites and uptake of pre-delivery uterotonic prophylaxis resulted in reductions in both the pool of delivering women and the actual incidence of PPH. Attempts to increase enrollment by adding 16 study sites in the Copperbelt region in 2008 did not result in increased clinic mean enrollment greater than 0.8 cases per clinic per month. (PDF) [file pone.0076477.s003.pdf]

Text S2: Low Accrual

The original estimate of the sample size was based on a pilot study we conducted in Nigeria in 2004-2006, with an adverse outcome rate of 9% [1] We used a 5% incidence of severe OH taken from the literature.[2-4] We applied what we thought was a conservative 4.5% incidence rate of OH to assessments of delivery rates and transfer rates occurring in Harare and Lusaka facilities in 2005. A sample size of 2400 women was calculated based on a reduction in incidence from 9.0% to 4.5% in extreme adverse outcome at 20 clinics, of varying sizes, 80% power, two-sided type 1 error rate of 5% and an intra-cluster correlation coefficient of 0.01.[5] Achieving this target required enrolling approximately 3.3 women per clinic per month over 3 years, which we felt was possible based on the assessment of delivery rates in Harare and Lusaka.

By the time data collection for the RCT began in 2009, two major events happened which affected both the overall pool of delivering women and actual incidence of PPH. The size of the population in Harare was reduced by the mass forced migration of shack dwellers in the peri-urban sites served by our primary health care clinics, Operation Murambatsvina, which affected more than 700,000 people.[6] The incidence rate of PPH in all sites decreased, perhaps due to the uptake of pre-delivery uterotonic prophylaxis by the midwives at the PHC level, which was not routinely performed during the assessment phase (2005). Prophylactic uterotonics have been demonstrated to decrease PPH by up to 60%.[7-9] By 2009, over 96% of all women delivering at PHCs received prophylaxis. Finally, as has been noted in other studies, participation in research trials, which emphasize standardized care and enhance data collection and recording of events has been shown to improve outcomes, even in the control arms.[10] In our study, there was an emphasis on prevention of PPH and training on the standardized protocol for management of PPH and prevention/management of shock; the clinical study staff participated in at least 3 training sessions (before each of the study phases), as well as frequent refresher trainings by research staff throughout the trial (Text S3, Table S1).

By 2008, when we were consistently below the expected rate of enrollment, we attempted to increase enrollment by adding the sites in the Copperbelt, 14 PHCs and two RHs, by increasing study monitoring of enrollment criteria, enrollment procedures and data forms, by continual retraining of low enrolling individual clinician/data collectors and PHCs, and by monitoring admissions to the RH for eligible patients sent from intervention or control clinics. However, these efforts never brought the per clinic mean enrollment to greater than 0.8 cases per clinic per month.

1. Hensleigh P, Ojengbede O, Miller S, Morhason-Bello IO, Fabamwo OA (2005) Non-pneumatic Anti Shock Garment Resuscitation in Patients with Post Partum Hemorrhage, Abstract # 029 Presented at the Society of Gynaecology and Obstetrics of Nigeria (SOGON) Annual Conference. Tropical Journal of Obstetrics and Gynaecology 22: S14.
2. Miller S (2006) Non-pneumatic Anti-Shock Garment for Obstetrical Hemorrhage, Study Protocol. University of California, San Francisco.
3. Carroli G, Cuesta C, Abalos E, Gulmezoglu AM (2008) Epidemiology of postpartum haemorrhage: a systematic review. Best Pract Res Clin Obstet Gynaecol 22: 999-1012.
4. Bais JM, Eskes M, Pel M, Bonsel GJ, Bleker OP (2004) Postpartum haemorrhage in nulliparous women: incidence and risk factors in low and high risk women. A Dutch population-based cohort study on standard (> or = 500 ml) and severe (> or = 1000 ml) postpartum haemorrhage. Eur J Obstet Gynecol Reprod Biol 115: 166-172.

5. Donner A, Klar, N. (2000) Design and Analysis of Cluster Randomisation Trials: In Health Research: Oxford University Press. 178 pages p.
6. Tibaijuko AK (2005) Report of the Fact-Finding Mission to Zimbabwe to Assess the Scope and Impant of Operation Murambatsvina by the UN Special Envoy on Human Settlements Issues in Zimbabwe.
7. Derman RJ, Kodkany BS, Goudar SS, Geller SE, Naik VA, et al. (2006) Oral misoprostol in preventing postpartum haemorrhage in resource-poor communities: a randomised controlled trial. Lancet 368: 1248-1253.
8. WHO (2012) WHO recommendations for the prevention and treatment of postpartum haemorrhage. Geneva: World Health Organization. 42 p.
9. Mobeen N DJ, Zuberi N, Jahan N, Blum J, Wasim S, Walraven G, Hatcher J (2010) Use of misoprostol by trained traditional birth attendants to prevent postpartum haemorrhage during home deliveries in Pakistan: a randomised placebo-controlled trial. British Journal of Obstetrics and Gynaecology.
10. Goudar SS, Chakraborty H, Edlavitch SA, Naik VA, Bellad MB, et al. (2008) Variation in the postpartum hemorrhage rate in a clinical trial of oral misoprostol. J Matern Fetal Neonatal Med 21: 559-564.
