# Supplementary material for: Non-Pneumatic Anti-Shock Garment (NASG), a First-Aid Device to Decrease Maternal Mortality from Obstetric Hemorrhage: A Cluster Randomized Trial
Source: PLoS One. 2013 Oct 23;8(10):e76477. doi: 10.1371/journal.pone.0076477 (PMC3806786; doi:10.1371/journal.pone.0076477)
Supplement: NIH Protocol S1 — The NASG RCT protocol. (PDF) [file pone.0076477.s005.pdf]

D. METHODS

D.1. Target populations

The main target population for the study intervention will be low-income pregnant, postpartum, and post-abortion women who develop severe obstetric hemorrhage and hypovolemic shock and who live in the selected Satellite Health Facility (SHF) catchment areas in Zambia and Zimbabwe.

D.2. Overall design

The study design will be a cluster randomized controlled trial to examine the effects of NASG application as a first-aid device at the Satellite Health Facility (SHFs) before transfer to Referral Hospitals (RHs).

The first step will include start-up activities and formative data collection, including facility staff training in data collection, how to collect blood in the closed-end blood collection drape, and in an evidence-based standardized clinical protocol for obstetric hemorrhage prevention and hemorrhage and shock management (See Table D.1). Next will be a period of baseline data collection at the RHs and SHFs during which clinical and demographic data will be collected from women diagnosed with obstetric hemorrhage and shock. After this baseline data collection period, we will introduce the study intervention, initially at the RHs and then at SHFs. The intervention will include: review of study protocol differences between baseline and the NASG-intervention phases, provision of the NASG, detailed training on the use of NASG for health care providers and staff, as well as on-site support and supervision for use of the NASG. After the RH providers are fully trained and have become proficient in NASG use, SHFs will be randomized into 13 intervention and 13 control facilities, as described in Section D.7 below. The final step will be three years of NASG-intervention data collection at the RHs and the SHFs on women diagnosed with obstetric hemorrhage and shock on the same outcomes collected in the baseline period.

Figure D.1. Design of Cluster Randomized Controlled Trial

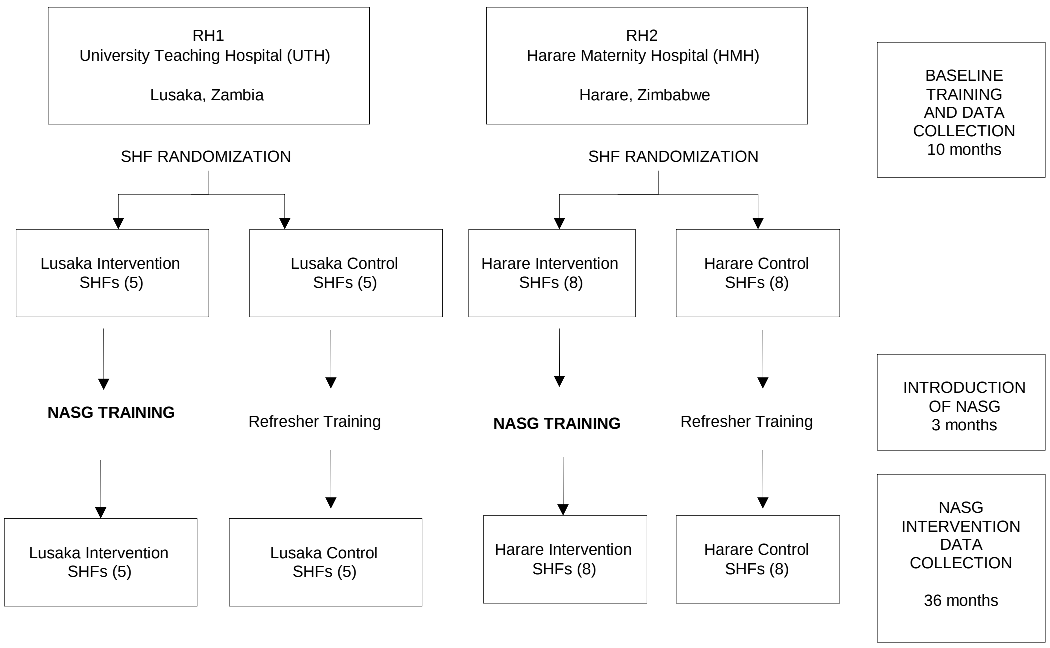

D.3. Justification for study design: Why is A Cluster Randomized Trial Necessary?

The study design is a cluster randomized trial to evaluate the effects of applying the NASG as a first-aid device to resuscitate and stabilize women suffering obstetric hemorrhage and hypovolemic shock at the SHF level before transfer to a RH.

The justification for a randomized trial is that there have been no randomized controlled trials of the NASG for any indication, and the only RCTs of the NASG's predecessor (4), the PASG, were inconclusive or actually showed worse outcomes. Although the case series from Pakistan (17, 18) and the pilot pre-post study in Egypt (1) seem to indicate that the NASG stabilizes and resuscitates women with obstetric hemorrhage and

hypovolemic shock, restores their vital signs, decreases blood loss; due to the weakness of the study designs, the small sample sizes, and lack of statistical significance on the crucial clinical outcomes (mortality and morbidity) there is not adequate evidence that the NASG does reduce extreme adverse outcomes. The current emphasis among the donor agencies and the Ministries of Health of low resource countries is on evidence-based reproductive heath care (117, 118). The weak evidence provided by the case series and pilot studies will not convince them to invest in procurement and training on an untested device. The PI of this proposal, Dr. Miller, has presented the current evidence at the WHO and the World Bank (See Appendix 8), each time she has been told that there is interest in the device, but, until there is strong evidence from an appropriately rigorous study in a low resource setting with high MMR, they will not accept it for use in their reproductive health programs (see Appendices 9 and 10). Further, when the article on the pilot study in Egypt (1) was reviewed by the BJOG, the reviewers were unanimous that : "This paper is of great clinical importance…. It is important that the potential for misleading results in an unblinded, non-randomised, before- and after trial be fully explored and stated. The promising results should be used as a basis of more methodologically robust studies, not for implementation in practice." (See Appendix 11)

Presently, since the efficacy of the NASG for decreasing morbidity and mortality is unknown, there is equipoise between best practice management of obstetric hemorrhage and hypovolemic shock and first-aid use of the NASG. As described section in B.7.a. there have been 3 poorly randomized, non-blind allocation RCTs of the PASG (69) ; to date there have been no RCTs demonstrating the effectiveness of the NASG for obstetric hemorrhage.

However, while a definitive trial of the NASG for obstetric hemorrhage in low resource settings might better be accomplished by the most unbiased of all designs, the randomized controlled trial of individual patients, there are some practical reasons why we have chosen the cluster design. Cluster randomized controlled designs (or group randomized trials) are appropriate when the allocation of identifiable groups to intervention and control arms of the study is necessary (119). Based on our experience and discussions with providers, we anticipate that it would be difficult for clinicians working at the same health facility to use different interventions on different women in such emergency situations as life-threatening hemorrhage. It is thus likely that randomizing individual patients would result in a considerable non-compliance with the study protocol, depending on the provider and the woman's situation. Having all providers in a single facility using the same basic intervention for women with severe obstetric hemorrhage is more feasible and more closely resembles the actual conditions in which the NASG would be used. Therefore, we will randomize allocation of the intervention at the SHF level.

Designing the study for use at the RH level only (after transport) was also considered. This is the method that was used in the pilot studies. While our experience in the pilots tells us that use at the RH level would probably affect resuscitation from shock and decrease bleeding, it is likely that the NASG will be placed too late to affect differences in mortality and morbidity (16, 19). Because the NASG is a first-aid device, the placement of the NASG at the SHF level in a randomized design has the best chance of demonstrating an effect on mortality and morbidity attributable to the earliest possible placement of the device (16).

Therefore, conducting the study as a randomized cluster trial of the effects on morbidity and mortality from early placement of the garment at the SHF level seems the strongest and most feasible study. Placing the NASG only at the intervention SHFs also avoids the problem of denying some women what appears to be a useful device by having all women, regardless of arm of the study or transfer from SHF, receive the garment on arrival at the RH. A DSMB (Section E 5 d) will examine the evidence at 9-month intervals, and stopping rules will be followed if the evidence becomes overwhelming that the NASG decreases extreme adverse outcomes.

D.4. Recruitment and enrollment of participants
D.4.a Study Sites
Zambia and Zimbabwe were selected for this study because they have high MMRs (Zambia, 729/100,000 live births (46), Zimbabwe between 695-1,100/100,000 (11, 47) with obstetric hemorrhage as the leading cause of death, high annual delivery rates of complicated cases in RHs, and a decentralized maternity care system wherein the majority of women who deliver in facilities do so at the SHF level. Indeed, in both Zimbabwe and

Zambia, potential sites for the proposed study, the Ministries of Health have asked for increased attention to the problem of maternal mortalities and morbidities (See Appendices 12 and 13). Both countries have university teaching hospitals with strong research infrastructures and experience in conducting clinical research. Thus setting the study in these countries is compelling substantively, in that we can make an impact on maternal morbidity and mortality, as well as practically and methodologically, in that the decentralized setting offers the opportunity to utilize a randomized cluster methodology to see the effect of the NASG during the transport period. In addition, the clinical personnel at the facilities and within the research departments at the universities in these countries are knowledgeable about and experienced in conducting clinical research.

Referral Hospitals in Zambia and Zimbabwe have been selected based on the following criteria: able to provide CEmOC (including blood transfusions and surgery), have at least 12,000 deliveries per year, be part of a decentralized and functioning referral system, and have at least 10 SHFs transporting their hemorrhage patients there. SHFs in Zambia and Zimbabwe will be selected based on the following criteria: able to provide basic emergency obstetrical care, but no capacity for blood transfusions or surgery, have at least 1,000 deliveries per year, and transfer complicated cases to a study RH.

D.4.a.1. Zambia

In Lusaka, population 1,500,000 people, there is one main public RH, the University Teaching Hospital (UTH), and one other maternity hospital, Chainama Clinic. All complications are referred to UTH, which has around 14,000 deliveries annually. There are 23 SHFs, the Lusaka District Clinics; of these 10 are midwifery-led delivery sites. The staffing at these SHFs are general practice physicians or university trained nurse midwives. Annual delivery rates at these SHFs range 650-4000, with a mean annual delivery rate of 1000. Each of the SHFs is approximately 15 km from the RH via the one functioning ambulance. The RH and the SHFs recently participated in a comprehensive needs assessment of Emergency Obstetric Care (111), which involved the completion of multiple study forms. A letter of support from the Director of Public Health and Research of the Zambia Central Board of Health (Victor Mukonoka) regarding the willingness and ability of the staff from these health facilities to participate in the proposed study is included in Appendix 12.

D.4.a.2. Zimbabwe

There are over 2,000,000 people in Harare and two public maternity Referral Hospitals, Harare Maternity Hospital (HMH), with 11,500 births in 2004 and Mbuya Nehanda Maternity Hospital, with 3,200 births. HMH receives referrals from 13 city council clinics, as well as receiving referrals from 3 nearby provincial and district hospitals. The RH level MMR is 400 /100,000, and no maternal deaths occur at the SHFs, as all complicated cases are referred to HMH. More than 30,000 deliveries occur annually in the 16 SHFs (city council clinics and provincial/district hospitals that refer to the HMH) in the Harare area, and each SHF is about 30 minutes by ambulance from HMH. Annual delivery rates at the SHFs are 1,000 or more births per facility. A letter of support from the City of Harare Director of Health Services (Dr. O.L. Mbengeranwa) regarding the willingness and ability of the staff from these health facilities to participate in the proposed study is included in Appendix13.

D.4.b Inclusion/Exclusion Criteria for Women

Eligibility criteria include: pregnant, postpartum or postabortion, diagnosis of obstetric hemorrhage from any cause (postpartum, post-abortion, ectopic pregnancy, ruptured uterus, placenta previa, abruption of placenta, placenta accreta, etc.), if ante-partum hemorrhage, have a nonviable fetus, estimated blood loss of > 750 mL, vital signs indicating shock (SBP < 100 mm Hg or pulse > 100 bpm), able to provide informed consent to enter or continue the study and for the study to use her data (See Section E), and willing to use the NASG as primary intervention until definitive treatment becomes available.

The following criteria are absolute exclusion criteria: current viable third trimester intrauterine pregnancy that can be delivered in the next 20 minutes after hemorrhage begins and/or current bleeding sites above the diaphragm. Relative exclusion criteria include: a history or current clinical evidence of mitral stenosis or congestive heart failure (CHF). These must be relative contraindications that will be assessed at the time of hemorrhage and clinical judgment will need to be utilized to evaluate each case individually. Thus, if the patient can be transported and delivered rapidly and is suspected or known to have either condition, she will be excluded from the study. In other cases, if the patient is dyspneic in the NASG at the SHF level, the NASG will be loosened, if that does not result in relief, the NASG will be removed. If the patients at the RH have

decreasing oxygen saturation levels as measured by pulse oximeter, the NASG will be loosened, if that does not result in relief, the NASG will be removed.

In order for the subjects and data to be independent, data on a second incident of severe obstetric hemorrhage from women who have already participated in the study (a subsequent pregnancy with recurrent hemorrhage), will be identified from medical record numbers and other identifying information recorded on separate forms held at the RHs in country. If these women consent to participate in the study they will receive treatment according to the study protocol, but their data will not be used in main study analyses evaluating the effectiveness of the NASG. Based on data on fertility and birth intervals in Zambia and Zimbabwe and on an estimated recurrence risk of 12.1% for postpartum hemorrhage and/or retained placenta (120), we estimate that we would have a maximum of 50-60 such cases in each country during the three-year NASG intervention data collection period. Our sample size calculation is conservative (see Section D.8) and the sample will be sufficient even if these numbers of women need to be excluded.

D.5. Start-up activities

During the first six months of the study, investigators will work with national and local governments, ministries of health, health care systems, research institutions, NGOs, and the participating health care sites in the two countries to build the necessary partnerships and develop a foundation of support for this study. Outreach to communities will be conducted by project-trained community outreach workers in order to obtain local approval and understanding of the study, including informational materials designed to be culturally appropriate and for the assessed level of literacy (See Appendix 19). Other start-up activities will include formative assessments at participating health facilities in order to determine characteristics of study sites prior to randomization, final selection of study sites, adaptation of the Case Report Forms (CRFs) and manual of operations used in the pilot study in Egypt, adaptation of training curricula used in Egypt (RH), Nigeria (RH and SHF), and Mexico (SHF) for intervention and control sites, training of study data management personnel, and training of study clinical personnel in evidence-based management of obstetric hemorrhage, as outlined in Table D.1 (42, 121).

D.5.a. Referral Hospitals and Satellite Health Facilities

Additional data on the characteristics of each study health facility will be obtained during the start-up phase. The purpose of this assessment is to assess the extent to which all RHs and SHFs are roughly equivalent in terms of facility characteristics and standard management of PPH, and to identify additional topics for the trainings that will be carried out in order to standardize practices. This assessment will occur during site visits using inventory checklists, record review checklists, observation checklists, and interview guides (106). We will focus on observations of the current management of obstetric hemorrhage and shock, in order to determine what changes will need to be made in order to revise protocols to reflect the best-practice evidence-based standardized care. Obstetric delivery logs and emergency room logs will be reviewed for admissions with obstetric hemorrhage; individual patient's record reviews for obstetric hemorrhage cases will be conducted in order to validate maternal mortality and morbidity statistics.

D.6. Baseline Data Collection

Immediately prior to baseline data collection, introductory meetings will be organized at each site to explain the purpose of the study, the study design, the potential of the NASG to decrease maternal mortality and morbidity, and the timeline for the study. These meetings will include trainings to familiarize all staff with the details of the study protocol, the organization, and management of the CRFs and to engage staff in working towards the study's goals. Essential will be training in the evidence-based best practices of clinical obstetric hemorrhage and shock treatment in order to upgrade skills and knowledge of all RH and SHF clinicians and update all facility protocols in order to ensure that all facilities are providing equivalent care. The baseline training will include both didactic and experiential methodologies.

Training in the baseline and pre-intervention phase will be led by Dr. Miller, who has 3 years experience in training physicians, surgeons, nurses, midwives, anesthesiologists, and village level health care workers to use the study CRFs and protocols appropriate to each level and type of health care provider. She will work together with Dr. Oladosu Ojengbede, who is the Principal Investigator of the Nigeria NASG case series and a consultant on the proposed study. Dr. Ojengbede will bring his expertise with the NASG, the study and protocols, and his real-world experience in low resource Sub-Saharan Africa health facilities. The pre-

intervention trainings will be conducted at each RH for 1 day of didactic training: review of training in active management of third stage labor (AMTSL), training and institution of evidence-based management of obstetric hemorrhage and shock, review of training in ethical considerations in research, question-by-question review of the long-form CRFs and manual of operations for completing the forms. The second ½ day will be spent in hands-on training in use of the blood collection drape and CRF completion.

At the SHF level, the team of Dr. Miller, Dr. Objengbede, and master trainers from the University of Zimbabwe and the University of Zambia will conduct trainings at 5-6 SHFs in each country. Staff from 2-3 SHFs will come to one SHF for the one day training in review of training in AMTSL, training and institution of evidence-based management of obstetric hemorrhage and shock, review of training in ethical considerations in research, question-by-question review of the short form CRFs and manual of operations for completing the forms. There will be a short hands-on session in placing the adult diapers prior to transport (See D7a). Supervisors from the Central Office, who will be trained at the RH level, will cover the shifts of the staff who will miss clinics to come to the training.

The baseline data collection will involve standardized data collection at each study site. Following a one-month trial run for piloting the CRFs and the study procedures, the baseline data collection period will begin and continue for a period of six months at the RHs and nine months at the SHFs. The rationale for the length of the baseline is to allow an adequate time for understanding clinical management issues and to acquire adequate data on which to reformulate sample size calculations using the most up to date and most accurate recording of mortalities and SAMMs. Globally, mortalities are often under-reported and SAMMs are rarely recorded or reported. We estimate that approximately 963 women (585 from the SHFs and 378 from the RHs) will be included in the baseline data collection.

The Case Report Form (CRF) for the SHFs is a 2-page form that will not demand more than the typical chart notations made on an obstetric hemorrhage case for transfer that is usually completed at the SHFs. These forms are adapted from the short forms used in the Mexico Pilot Study by low level village health workers (81). Our experiences there demonstrate that this level of data collection can be managed even in such facilities. The health care workers, particularly nurses and nurse-midwives, at the SHFs are trained in research during their pre-service training, and they routinely participate in data collection (111, 113, 122). The only new information requested on the CRFs is the time of the start of the hemorrhage, documentation of the patient's permission to use the data in the study, time of placement of the adult diapers, and a recording of the patient's hemoglobin on admission to the study. The hemoglobin will be assessed with portable hemoglobin analyzers (Hgb Pro Professional Hemoglobin Testing System™) placed at every SHF. After collecting data on the woman's condition at entry to the study at the SHF level, the patient will be transferred to the RH, where the bulk of the data collection will occur. (See Appendices 14 and 15)

The baseline RH CRF will record information on individual patients with a diagnosis of hypovolemic shock secondary to obstetric hemorrhage (for the purposes of this study, criteria include blood loss > 750 mL, systolic blood pressure < 100 mm Hg or pulse > 100 beats per minute). The collection of data will be prospective, continuous, and observational. CRFs used for the pilot studies will be revised for this expanded study. CRFs for each country will be adapted in consultation with medical staff in the health systems within each country following a review of their medical record formats. Forms will be filled out by the health care provider managing the case, with support from the on-site study coordinator.

Data to be collected at the RH level will include: data on admission to study if patient did not come as a referral from the SHF; time to recovery of consciousness; time to return to normal pulse; initial lab results (hemoglobin, creatinine, and type and cross match); weight of adult diapers if used in transport from the SHF; results of tests for coagulopathy, if indicated; flow chart for recording pulse, blood pressure, volume of measured blood loss in the blood collection drape, measured urine output, and oxygen saturation at regular intervals; flow chart information on volume of blood transfused and volume and type of IV fluids infused; type, amounts, route of administration and times of uterotonic administration; surgical and non-surgical procedures performed; hemoglobin and creatinine results at study discharge; hospital discharge lab results; definitive diagnoses; information on whether transfer to an intensive care unit was required; survival outcomes of the patient; and extreme adverse outcomes.

D. 7. Intervention

The study intervention will consist of detailed training on the use of NASG for health care providers, ancillary health care staff, emergency medical technicians, facility laundry and cleaning staff, and ambulance drivers, followed by continuous support and supervision for use of the NASG. Efforts will be made to ensure that every individual from the selected health facilities (who may need to use the NASG at some point) is present during the full training, and that additional trainings will be provided to those who cannot attend. The trainings will again be separate for RHs and SHFs. The 1-day training at intervention SHFs will consist of: a review of the elements of the baseline training listed above in D.6**,** overview of intervention study protocol**,** question-by-question review of changes in the CRFs relative to the use of the NASG (approximately 6 new entries required over baseline and control forms, see D.9), and hands-on, step-by-step training session on the application of the NASG, role-play practice putting the garment on both conscious and unconscious "patients," and application of the adult diaper with the NASG. Although we anticipate NASG removal to be rare at the SHFs, we will train in proper removal. The trainings will again be held at 5-6 SHFs in each country, with staff coming from 2-3 SHFs to each training. Control SHFs will continue to manage women with obstetric hemorrhage according to the same evidence-based clinical protocol as that used at intervention units, except for the use of the NASG. Staff at the control SHFs will receive a refresher training on the same curriculum they received before baseline collection, plus problem solving exercises regarding real-life problems that have arisen during the baseline period. These trainings will be held at the same time the intervention clinics receive their NASG training so that both control and intervention sites have the same level of reinforcement for study and clinical practices. Supervisors from the central health office, who have attended trainings at the RH level will provide training at the SHFs for those who were not able to attend the larger trainings.

The RH trainings, held at each RH, will be 2 days and consist of the same didactic training as the SHFs, replacing the use of the adult diapers with the use of the blood collection drapes. The second day of training will focus on receiving patients in the NASG, application of the NASG, use of the NASG during surgery, and on the correct removal of the NASG. This training will be repeated once for RH staff who could not make the first training, and those who were unable to attend either session will be trained separately by the study-trained supervisors.

After the RHs receive this training, there will be a one-month pilot run using the NASG and the Case Report Forms before the official NASG-intervention data collection begins. Study sites will be visited at least monthly, depending on the volume of hemorrhage cases, by members of the study team who will monitor use of the NASG and data collection, supervise health care providers regarding the study protocol, and provide additional training and support for use of the NASG. The staff at the RHs will use the NASG for 3 months before they start receiving transported patients in the NASG from the SHFs, so that they become proficient in the use of the garment at their facilities. All women who arrive at the RH, regardless of randomization arm or transfer from SHF, will be placed in the NASG, as we have indicated in Section D.3. Based on data from previous pilot studies in Egypt (1), we do not believe that it would be practical to ask providers at the RH to withhold placing the NASG on hemorrhaging patients arriving from the control SHFs.

D. 7. a. Table D.1: NASG Clinical Protocols :

Key: ◊ indicates procedures done in both the control and study groups that are study specific,<br>shaded rows show procedures that will be done in NASG group only<br>** are procedures that would be performed for all hemorrhage patients, even patients who do not enroll in the study<br>SHF and RH indicate at which level of health care facility the activity is performed		
**1.<br>SHF<br>RH	Prevent PPH	Prevent PPH in all patients, by the active management of the third stage of labor, which has been shown to decrease the incidence of PPH (50). Administer 10U IM oxytocin with the delivery of the baby, deliver the placenta with a contraction and by controlled cord traction, and rub the uterus after delivery of the placenta to be sure it is firm.
**2.<br>SHF<br>RH	Diagnose Hypovolemia	Hypovolemia for this study is: blood loss > 750 mL and either, SBP <100 mm Hg or pulse >100 bpm.
**3.<br>SHF<br>RH	Give Oxygen	Give oxygen by mask at 10 liters/minute, and, if the woman is unconscious, maintain the airway, suction, and reposition as needed to prevent aspiration and to assure normal ventilation
4.	Place NASG	Lie the patient on the NASG, start at the ankle section #1 and close each segment,

Inter-vention SHF & RH	(NASG Intervention Phase Only)	sequentially, and as tightly as possible. Two people can rapidly close the 3 leg segments, but only one provider should close segments 4 and 5. For PPH, immediately before closing segments 4 and 5 massage the uterus and expel any clots or blood pooling in the vagina. Estimate and record this amount on the flow chart.
◊5. SFH RH	Place Drape or Diaper to Measure Blood Loss	*SHF: W*omen should be sent to the RH as rapidly as transportation can be arranged*. Blood loss measurement during transport:* Place an adult diaper, which is less likely to fall off during transport than the blood collection drape. The diapers will have been pre-weighed dry and can hold 2000 mL before reaching saturation. Upon arrival at the RH, remove and then re-weigh the diaper. Place the weight on the CRF.<br><br>*RH: Use of the blood collection drape*: Place the top blue section of the drape under the woman's buttocks with the lower portion (pouch) remaining open to collect the blood. Measure blood loss using the graduated mL marks on the pouch. Measurements should be recorded every hour in the acute phase of the hemorrhage, and then every 4 hours. Change drape at least every 4 hours.
**6. SHF RH	Replace Fluids	SHF: Begin IV of Ringer's Lactate or Normal Saline with large bore IV catheter. Rapidly infuse 1500 mL in one IV. If the trip to the hospital is longer than 30 minutes, bring 2 more containers of IV fluids of 1000 mL, and infuse enough to keep BP at 80 and pulse below 140.<br><br>RH: Start two large bore IV catheters. Until blood transfusions can be arranged, and surgical intervention provided as needed, women will receive a limited infusion of intravenous saline. Rapidly infuse 1500 mL of normal saline in one IV. For women with uterine atony, start another liter of saline with 20 Units of oxytocin, run at a rate of 150 mL/hour. Give bolus infusions of 500 mL normal saline (without oxytocin) as rapidly as needed to maintain a systolic blood pressure of at least 80 mm Hg and pulse below 140 bpm. As soon as blood is available for transfusion and/or surgical intervention is initiated, the rate of infusion may be increased up to 250 mL/hour with bolus infusions of 500 mL (without oxytocin) given as needed to maintain the systolic pressure above 100 mm Hg. Bolus IV fluids will be limited to 5,000 mL total intravenous crystalloid solutions in the first 6 hours and 8,000 mL in the first 24 hours.
**7. SFH RH	Monitor Fluid Status	As soon as airway and IV access are addressed, and diagnostic evaluation is complete, place a Foley catheter to dependent drainage with a measuring device connected for documenting hourly urine output. Document input of fluids and output of urine at least hourly during the resuscitation and then every 4 hours until blood replacement is complete and there is no further bleeding.
**8. SHF RH	Laboratory Tests	SFH: Obtain finger stick Hgb at time of admission to study.<br>RH: Order a minimum of CBC, creatinine, blood type, and cross match for 4-6 units blood transfusion (or more as required). Any clinical evidence of coagulopathy (initially or any time during treatment) should be further revaluated with tube clot observation, platelet count, and bleeding time. Before each blood transfusion and prior to removing the NASG, hemoglobin tests will be repeated. Prior to hospital discharge, Hgb and creatinine (if elevated during the study) will be repeated. Other tests should be ordered per standard hemorrhage management.
**9. SHF RH	Patient Evaluation for Source of Blood Loss	As soon as the initial resuscitative measures are underway and another attendant is available, determine the specific site of blood loss. Examine the patient vaginally to identify and rapidly repair any lower genital tract lacerations. Manage women with uterine atony as below in #10. If there is evidence of retained tissue, perform manual removal, manual vacuum aspiration, and/or curettage.
**10. SHF RH	Manage Uterine Atony	First attempt: vigorous fundal massage, then bimanual compression<br>Uterotonics:<br>First 10 minutes: rapidly infuse oxytocin solution 20 IU/liter of normal saline; continue uterine massage and compression until uterus becomes firm. Give Ergometrine, 0.5 mg IM, or misoprostol 800-1000 micrograms rectally.<br>Second 10 minutes: continue massage of uterus and infusion of oxytocin up to completion of 1000 mL total, and then reduce to 20 units of oxytocin per liter at 150 mL/hour
**11 RH	Blood Transfusions	Arrange blood transfusions per hospital protocol
**12. RH	Surgery	If laparotomy is required, determine when the patient is sufficiently stabilized and sufficient blood replacement available to allow for surgical intervention. Immediately after opening the abdomen, suction all hemoperitoneum. Measure and record the volume, then change the suction bottle and record the remainder of blood suctioned during the surgery as

		"intraoperative" blood loss. Surgical procedures could include any method to stop bleeding, as appropriate, including uterine artery ligation, iliac artery ligation, B-Lynch or other compression suture of uterus, evacuation of molar pregnancy, salpingectomy, salpingostomy, or hysterectomy.
**13. RH	Manage Oliguria (< 30 mL per Hour for > 4 hours)	Evaluate for adequate fluid intake, if fluids have not exceeded the limits of 5,000 mL for the first 6 hours or 8,000 mL for the first 24-hour period, an additional bolus of 500 mL may be given and the output assessed over the next hour. Alternatively, or if the patient fails to respond to the bolus, give an infusion of low dose dopamine (5 micrograms/kg/hour) using a separate intravenous line.
14. RH	Remove NASG	Once the patient has been stabilized, with no bleeding greater than 50 mL/hour, normal vital signs for two hours, and Hgb ≥ 7.5, begin removal of NASG. Start at the ankles with segment 1, open, wait 15 minutes, and take pulse and blood pressure. If there is no change, continue one segment at a time, taking pulse and blood pressure 15 minutes after opening each segment before moving on to the next segment. If the BP drops by 20 mm Hg OR the pulse rises by 20 bpm, reclose the opened segment (s), increase IV fluids and try to determine if bleeding has resumed.
◊15. RH	Remove blood collection drape	Once the patient has been stabilized, with no bleeding greater than 50 mL/hour and normal vital signs for two hours, and Hgb ≥ 7.5, remove the NASG and then the blood collection drape.<br>For pre-intervention patients, remove the blood collection drape once the patient been stable for two hours, with no bleeding greater than 50 mL/hour and Hgb ≥ 7.5

D.8. Sample description and size

In order to assure that the study has sufficient sample size to detect changes in a clinically significant outcome, the sample size calculations for the proposed study are based on an estimated reduction in the proportion of women with obstetric hemorrhage who die or experience severe morbidity (together referred to as "extreme adverse outcomes"). A sample size calculated in this manner also gives us high power to detect differences in other key variables, such as volume of blood loss.

The incidence of obstetric hemorrhage leading to hypovolemic shock is around 1-3% of deliveries in developing country settings [1, 2], and likely to be even higher than 3% in high maternal mortality countries such as Zambia and Zimbabwe. Based on recent data from various low-resource settings in Africa, we used an estimated incidence of severe obstetric hemorrhage (>= 1000 mL) of 4.5% for the satellite health facilities (SHFs) and 7% for the referral hospitals (RHs) that receive more emergencies and complicated cases, for our sample size calculations. In combined data on severe obstetric cases from the Egypt and Nigeria NASG pilot studies, a 72% reduction in extreme adverse outcomes was observed for NASG cases, as compared to non-NASG; 5 women (3.3%) who received the NASG versus 12 women (11.8%) who did not (RR = 0.28, 95% CI: 0.10---0.77)[3]. An estimate of the expected level of extreme adverse outcomes for the proposed study was made using these data from Egypt and Nigeria, as well as data on maternal mortality and morbidity from the University Teaching Hospital in Zambia [4, 5]. For the sample size calculation for the cluster randomized trial we estimated a level of 11.8% of obstetric hemorrhage cases ending in extreme adverse outcomes in the control group, and specified that we want to be able to detect a reduction of 50% (or more) in this outcome, to 5.9% in the NASG group.

The sample size for the cluster randomized trial was calculated using the methods described by Donner and Klar [6] and using the ACLUSTER Software for the Design and Analysis of Cluster Randomized Trials [7] (Version 2.0.) A conservative estimate of the intra-cluster correlation coefficient (ICC) for the proportion with mortality or severe morbidity (.01) was calculated from the Egypt NASG pilot study data.

Additional assumptions for the sample size calculation include the following: If each SHF has a minimum annual volume of births of 1,000, then we can expect a minimum of 45 cases per SHF per year (assuming 4.5% of all obstetric patients develop severe hemorrhage). In 24 months, we can expect an average of 90 cases per SHF. Between the two countries we will have a minimum of 20 SHFs, 10 in each arm participating in the study. Thus, using the power calculation formula for an unstratified cluster design, we will have 89% power to detect a 50% reduction in extreme adverse outcomes (from 11.8% to 5.9%) at the 95% confidence level. If 90 women with obstetric hemorrhage leading to hypovolemic shock are observed in each cluster over the 24-month period after implementation of the NASG intervention, a total of approximately 1,800 women will be

included in the cluster randomized trial (20 x 90), 900 women in the intervention group and 900 women in the control group.

For cluster randomized trials with repeated cross-sectional binary measurements it is important to attempt to achieve baseline balance by stratification of the clusters prior to randomization [8]. In order to ensure balance between the two study arms in terms of important country and health facility characteristics, the sample will be stratified by country/referral hospital. In a stratified design, health facilities are grouped into homogeneous strata and then are randomly assigned, within each stratum, into the intervention or the control group [9]. In this case, the SHFs associated with each country/RH will be randomized to the intervention or the control group. In each stratum, random permutations will be produced using a SAS random number generator with the starting number taken independently for each stratum. Stratification in a cluster randomized design is likely to result in a reduction in the needed sample size or an increase in the power for a given sample size. Accounting for stratification by country/ RH (2 strata) in the sample size calculation, with a total of 1,800 women (20 SHFs x 90 women observed per SHF), a two-sided test, an alpha level of .05, an ICC of .01, and a constant odds ratio of 2.0 (50% reduction), increases our power to detect a significant difference between study arms to 92%. Based on the results of the initial assessment of health facility characteristics in the start-up phase of the study, the minimization allocation method [10] may be used in the randomization process to better achieve balance between the treatment groups.

Data on women with severe obstetric hemorrhage who are admitted directly to a RH (not transferred from a SHF) during the first three months of the NASG-intervention period will also be compared with data from similar women who were admitted directly to a RH during the three-month baseline data collection period. The estimated sample size for this pre-post comparison is 892 women (446 women during each phase), based on annual delivery volumes of 11,400 for the RH in Zimbabwe and 14,000 for the RH in Zambia, and an estimated severe hemorrhage rate of 7% for these referral facilities. This sample size will give us 88% power to detect a 50% (11.8% to 5.9%) or larger difference in extreme adverse outcomes for this population. This comparison is planned in order to allow us to confirm the results obtained from the pilot study conducted at relatively high resource referral hospitals in Egypt, in the very low resource settings of Zambia and Zimbabwe, with a larger sample size.

After the baseline data collection phase of the study (3 months at the RHs with an expected 446 cases, and 6 months at the SHFs with an expected 450 cases), the assumptions used to determine sample size will be assessed by the Data Safety Monitoring Board (DSMB), and any recommended alterations will be made to the study design. Based on our current assumptions, the total estimated number of cases to be enrolled in the study is 3142--896 cases during the baseline phase and 2246 cases during the NASG-intervention phase.

D.9. NASG-Intervention Data collection

NASG data collection will begin after the intervention trainings and 1-month trial runs of the intervention CRFs and NASG procedures have been completed, and continue for a period of three years in both intervention and control sites. As in the baseline, the bulk of data collection will occur at the RH level. NASG-intervention CRFs for intervention sites will gather information on all of the variables collected during the baseline phase, as well as the following: time/date NASG applied/removed, complications/side effects related to use of the NASG including need to remove NASG due to severity of side effects, recurrent bleeding or change in vital signs during/after removal of the NASG, and if patient has surgery while in NASG: time/date of opening of NASG abdominal and pelvic panels for surgery and time/date of closing of NASG abdominal and pelvic panels after surgery.

D.10. Measures

The goal of NASG use is to reduce the MMR from obstetric hemorrhage in low resource settings. However, studies to measure maternal mortality interventions are difficult, expensive and require very large samples (29). For this reason, the main outcome measure for this study will be the numbers of women with SAMMs or mortality, a combined measure we have called "extreme adverse outcomes." The incidence of SAMMs or "near miss events" is an important indicator related to maternal mortality (24, 123). Emergency hysterectomy is another extreme outcome that may be reduced with use of the NASG. In addition, the volume of blood loss as

measured by the blood collection drape is an important indicator of the effectiveness of the NASG. To obtain a relatively objective measure of blood loss, maternal bleeding after admission to the study will be measured using a specially designed, closed-end, calibrated plastic blood collection drape. Studies of this calibrated blood drape indicate that it is more accurate than visual assessment in measuring postpartum blood loss (131). Blood loss during surgery, "intraoperative blood loss" is likewise an important indicator, and due to the difficulty in determining how much blood was in the abdomen prior to the surgery (hemoperitoneum) and how much blood may be shunted to the abdomen during surgery (due to the shunting effects of the leg portions of the garment which are left in place), determining the amounts of blood lost during surgery becomes very important. Blood loss during surgery will therefore be measured twice, immediately upon opening the patient (to suction and measure hemoperitoneum) and then during the remainder of the surgery. It is crucial to discover if the decrease in blood loss in the drape with the NASG is offset by increased intraoperative blood loss, due to the shunting of blood from the extremities to the abdomen when the abdominal section is removed for surgery. In addition, data on other possible negative effects and any adverse reactions to the use of the NASG will be collected in order to determine whether or not there are negative side-effects related to NASG use. Due to the difficulty of locating women after they leave the hospital, all outcomes will be measured only up to the time that the woman is discharged from the hospital or pronounced dead in the hospital.

D.10.a. Main Outcome Indicators:

Table D.2 Study Definitions for Main Outcome Indicators

Indicator	Study Definitions
Number of immediate maternal mortalities from obstetric hemorrhage	Death of the subject after termination of pregnancy, while still in the hospital, from causes directly or indirectly the result of obstetric hemorrhage. This study will not be able to follow women after hospital discharge.
Number of diagnosed severe acute maternal morbidities (kidney disease, acute respiratory distress syndrome, cardiac deficiency, or central nervous system damage)	Occurring before discharge from the hospital:<br>a) Acute renal failure (oliguria, <120 mL output in 4 hours interval, serum creatinine >1.5 or increased >1.0 above baseline).<br>b) Acute respiratory distress syndrome (impairment of respiratory function requiring oxygen supplementation, ventilation, or limiting physical activity compared to pre pregnancy status)<br>c) Cardiac deficiency (impairment of cardiac function resulting in a change versus pre pregnancy cardiac status by New York Heart Disease Class (132)<br>d) Evidence of cerebral impairment (seizure, unconsciousness, motor or cognitive loss)
Number of Extreme Adverse Outcomes	Combined indicator of maternal mortalities and severe organ failure morbidities (SAMMs).
Time to Recovery from Shock	Time to improvement of level of consciousness from unconscious to conscious or from confused to normal by use of the Glasgow Coma Scale (87). Time to restoration to Shock Index less than 0.7 (86, 133), and time to achieve a MAP of ≥60 (2/3 * Diastolic BP) + (1/3 * Systolic BP) or as recorded on the pulse oximeter)
Change in hemoglobin level	Difference in hemoglobin level (Hgb) at point of entry to the study, at study discharge, and on hospital discharge
Number of emergency hysterectomies performed	Documented operative record of removal of the uterus
Volume of blood lost from the vagina	Blood loss after enrollment in the study as measured in mL using the closed-end blood collection drape (RH) and /or the adult diapers (SHF and during transport)
Volume of blood lost during surgery	If surgery is performed in order to differentiate between hemoperitoneum (blood already in the abdomen) and blood lost during surgery, the blood in the abdomen will be immediately suctioned into a graduated suction bottle and the measured amount will be recorded, then the bottle will be changed and subsequent blood lost during the surgery will be measured and recorded.
Other negative effects, possibly related to use of NASG	a) decreased urine output, b) hypoxia (SpO_2 <=83.5%) (134), dyspnea (a clinical sign of hypoxia that manifests as shortness of breath resulting in an increase in respiration rate and depth; sometimes also see exaggerated respiratory effort, use of accessory muscles, nasal flaring (135).), or other form of respiratory distress, and/or c) nausea and vomiting.

D.11. Analytic procedures

Analyses of data from the SHFs, given the randomized cluster design, will mainly be conducted at the cluster (SHF) level. Individual level analyses will take into account cluster effects (136). Primary analyses will be based on the "intention to treat" (ITT) principle, with facilities in the intervention group classified as "intervention" regardless of whether or not the NASG was actually applied to participants. The ITT approach is the most rigorous way to analyze data from randomized controlled trials and failure to analyze in this manner can give "misleading and indeed life-threatening interpretations" (137). Per-protocol analysis is likely to result in a biased estimation of the effect of the intervention, since eligible women at intervention sites who do not

receive the NASG may be different (more severe or less severe cases) than those who do receive the NASG. However, a per-protocol analysis will also be conducted for comparison with the results obtained from the ITT analysis. In addition, training at intervention sites will stress the importance of using the NASG for all women who meet the study entry criteria, without exceptions. The NASG will not be available at the control sites, so such protocol violations will not be an issue there.

Preliminary analyses for Aim 1 will be conducted to compute study outcome variables (frequency of extreme adverse outcomes, volume of blood loss, frequency of emergency hysterectomies and time to recovery from shock), to summarize characteristics of control and treatment sites to assess the effectiveness of randomization. Analysis of the primary outcome (site-specific proportions of combined mortality and severe morbidity) will be based on a comparison of proportions between study arms using a weighted t-test that accounts for correlations between individual outcomes within sites (138). Even though there are only 13 health facilities in each study arm, each mean or percentage will be based on a large number of births, and the normal approximation to the binomial distribution can be used (139). These tests will be evaluated based on a two-sided alternative hypothesis and 5% type I error rate. The same approach will be used for secondary outcomes (frequency of emergency hysterectomies, mean volume of blood loss and time to recovery from shock). Per-protocol analyses will be conducted using similar methods, with site-specific outcomes based only on "compliant" participants, those who actually received the assigned treatment as specified in the study protocol. Differences between effects detected in these analyses and those conducted under the ITT approach will be helpful in evaluating the efficacy of the treatment in situations where compliance is ideal.

Additional analyses for Aim 1 will be based on individual responses to the outcomes specified above and will use generalized linear random effects regression methods (140) to control for correlation of individual outcomes within sites. These analyses will focus on detecting time trends in the effects of treatment, differences in responses across countries, and individual characteristics related to responses to treatment (e.g. diagnosis of cause of hemorrhage, maternal age, parity, gestational age at delivery, onset of labor, and use of uterotonics). Results will be summarized as odds ratios for models based on binary responses (e.g. occurrence of an extreme adverse event), and mean changes for continuous responses (e.g. volume blood loss). Models assessing time trends will include chronological time of outcome occurrences, and summarize results in terms of slopes for average monthly changes in outcome means/proportions (overall, between arms and by site). Nonlinearity in time changes will be diagnosed with regression spline methods (141). For "time to recovery," survival analysis will be used. Event time analyses of time to recovery from shock will control for clustering by recruitment site using proportional hazards regression including a random effect term to provide valid estimates of variability (142). Additional random effects regression models will be fitted, including measured baseline outcomes from the six-month pre-randomization data collection period to evaluate changes in primary and secondary outcomes from baseline levels. These models will be similar in structure to those just described, with changes from baseline, assessed using contrasts comparing average responses at subsequent (post-randomization) time periods (in six-month intervals) to baseline levels. Analyses for the second aim will mirror those described above for Aim 1. Table D.3 summarizes the aims, main variables, and the basic analysis plan for each.

Table D.3

Aim	Main Variable(s)	Basic Analysis Plan
**Aim 1**	**Primary Outcome:**<br>Frequency of mortalities and frequency of severe morbidities combined as extreme adverse outcomes<br><br>**Secondary Outcomes:**<br>Mean amount of blood loss in mL as measured by the blood collection drape, frequency of emergency hysterectomy, time to recovery from shock	• Weighted t-tests to compare primary and secondary outcomes for intervention vs. control arms at the cluster level.<br>• Individual level analyses (random effects regression models) that take into account the cluster effects.<br>• Random effects regression analyses of change from baseline levels in primary and secondary outcomes in intervention vs. control arms.
**Aim 2**	**Negative Effects:**<br>Mean amount of urine output, frequency of hypoxia or other form of respiratory distress, mean amount of intraoperative blood loss, frequency of reports of nausea, frequency of vomiting episodes	• Weighted t-tests to compare negative effects for intervention vs. control arms at the cluster level.<br>• Individual level analyses (random effects regression models) that take into account the cluster effects.<br>• Random effects regression analyses of change from baseline levels in negative effects in intervention vs. control arms.

Data on women with severe obstetric hemorrhage who are admitted directly to a RH (not transferred from a SHF) during the first six months of the NASG-intervention period will also be compared with data from similar women who were admitted directly to a RH during the six-month baseline data collection period at the RHs. Baseline and NASG-intervention women will be compared in terms of the primary study outcomes (mortality, morbidity, blood loss, emergency hysterectomy, time to recovery from shock, negative effects, etc.). This will allow us to confirm the results obtained from the pilot study conducted at relatively high resource referral hospitals in Egypt in the very low resource settings of Zambia and Zimbabwe, with a larger sample size.

The ACLUSTER software program (Version 2.0) for the Design and Analysis of Cluster Randomized Trials (127) developed by UNDP/UNFPA/WHO/ World Bank Special Programme of Research, Development and Training in Human Reproduction of the World Health Organization, as well as SAS (143) will be used for analyses. A detailed analysis plan will be prepared following the initial health facility assessments and will be revised following preliminary analyses of the baseline data. This plan will be completed and reviewed prior to randomization of study sites.

D.12. Quality control and data management

Each country participating in the study will have a Study Director, one or more Project Coordinator(s), and Supervisor/Trainers who also provide support and supervision on NASG use, and a Data Manager. Each study site will name one senior staff clinician to function as the as on-site Study Coordinator. Study Directors will be highly qualified researchers (physician, nurse-midwife, or MPH public health specialist) with experience in complex studies and data collection in the area of maternal health. These persons will have overall responsibility for all aspects of the study in their country, including implementation of the intervention, support and supervision, data collection, data processing, etc. Project Coordinators will be mid-level researchers (physician, nurse-midwife, or MPH) who will be responsible for the day-to-day coordination of study activities in their countries. Trainers will be obstetrical clinicians proficient in training methods and in the prevention and management of obstetric hemorrhage. They will conduct the trainings at intervention and control sites and provide on-going support and supervision.

D.13. Time line

The total duration of the project is 5 years, including start-up activities, the baseline data collection, the intervention, and NASG-intervention data collection, as outlined below in Table D.4.

Table D.4.

**I. Start-up Activities (6 months)**	1. Health facility pre-selection<br>2. Collection of formative data at health facilities, including facility needs assessment for equipment and supplies, review of clinical protocols, and observations of clinical management of obstetrical hemorrhage<br>3. Adaptation of Case Report Forms and manuals of operations<br>4. Adaptation of training curriculum for intervention and control sites<br>5. Training of study data entry and management personnel<br>6. Training of study clinical personnel in evidence-based management of obstetrical hemorrhage
**II. Baseline Data Collection (7-10 months)**	1. One-month trial run of CRFs and procedures for baseline data collection<br>2. Data collection on management and outcomes for obstetric hemorrhage cases at both RHs (6 months) and satellite clinics (9 months)<br>3. Data entry and analysis (on-going)<br>4. Finalization of intervention training curriculum<br>5. Monitoring and supervision of implementation of standardized hemorrhage and shock treatment protocols.
**III. NASG Introduction at RHs (3 months)**	1. Hospital presentations on findings of baseline study<br>2. Review of research and evidence-based best practice hemorrhage and shock management<br>3. NASG training for health care providers at RHs<br>4. Review of CRFs and changes required for NASG-intervention data collection<br>5. One-month trial run of CRFs and procedures at RHs for NASG-intervention data collection
**IV. NASG Introduction at SHFs (3 months)**	1. Randomization of SHFs into intervention and control groups<br>2. Review evidence-based obstetrical hemorrhage management<br>3. NASG Training at intervention sites and Refresher Training at control sites<br>4. Review CRFs and make changes necessary for including NASG-specific data<br>5. One-month trial run of NASG-intervention study procedures at SHFs

**V. Data Collection at RHs and SHFs (36 months)**	1. Data collection on outcomes for obstetric hemorrhage patients admitted directly to RH (6 months)<br>2. Data collection on outcomes for obstetric hemorrhage cases transferred from SHF to RH (36 months)<br>3. Support and supervision for use of the NASG and protocol adherence at SHFs and RHs<br>4. Data entry and analysis (ongoing)
**VI. Final Analyses and Report writing (5 months)**	
