# Supplementary material for: Non-Pneumatic Anti-Shock Garment (NASG), a First-Aid Device to Decrease Maternal Mortality from Obstetric Hemorrhage: A Cluster Randomized Trial
Source: PLoS One. 2013 Oct 23;8(10):e76477. doi: 10.1371/journal.pone.0076477 (PMC3806786; doi:10.1371/journal.pone.0076477)
Supplement: Related Article S1 — A related article published in PLOS ONE. (PDF) [file pone.0076477.s006.pdf]

OPEN ACCESS Freely available online

PLOS | ONE

Use of the Non-Pneumatic Anti-Shock Garment (NASG) for Life-Threatening Obstetric Hemorrhage: A Cost-Effectiveness Analysis in Egypt and Nigeria

Tori Sutherland[1], Janelle Downing[2]*, Suellen Miller[3], David M. Bishai[4], Elizabeth Butrick[3], Mohamed M. F. Fathalla[5], Mohammed Mourad-Youssif[6], Oladosu Ojengbede[7], David Nsima[8], James G. Kahn[2]

1 University of Texas Southwestern Medical Center, Dallas, Texas, United States of America, **2** Philip R Lee Institute for Health Policy Studies, University of California San Francisco, San Francisco, California, United States of America, **3** Department of Obstetrics, Gynecology and Reproductive Sciences, Bixby Center for Global Reproductive Health and Policy, University of California San Francisco, San Francisco, California, United States of America, **4** Department of Population, Family, and Reproductive Health, Johns Hopkins Bloomberg School of Public Health, Baltimore, Maryland, United States of America, **5** Department of Obstetrics and Gynecology, Faculty of Medicine, Assiut University Women's Health Center, Assiut, Egypt, **6** Department of Obstetrics and Gynecology, El Galaa Maternity Teaching Hospital, Cairo, Egypt, **7** Department of Obstetrics and Gynecology, University College Hospital, Ibadan, Nigeria, **8** Department of Obstetrics and Gynecology, Katsina General Hospital, Katsina, Nigeria

Abstract

Objective: To assess the cost-effectiveness of a non-pneumatic anti-shock garment (NASG) for obstetric hemorrhage in tertiary hospitals in Egypt and Nigeria.

Methods: We combined published data from pre-intervention/NASG-intervention clinical trials with costs from study sites. For each country, we used observed proportions of initial shock level (mild: mean arterial pressure [MAP] >60 mmHg; severe: MAP ≤60 mmHg) to define a standard population of 1,000 women presenting in shock. We examined three intervention scenarios: no women in shock receive the NASG, only women in severe shock receive the NASG, and all women in shock receive the NASG. Clinical data included frequencies of adverse health outcomes (mortality, severe morbidity, severe anemia), and interventions to manage bleeding (uterotonics, blood transfusions, hysterectomies). Costs (in 2010 international dollars) included the NASG, training, and clinical interventions. We compared costs and disability-adjusted life years (DALYs) across the intervention scenarios.

Results: For 1000 women presenting in shock, providing the NASG to those in severe shock results in decreased mortality and morbidity, which averts 357 DALYs in Egypt and 2,063 DALYs in Nigeria. Differences in use of interventions result in net savings of $9,489 in Egypt (primarily due to reduced transfusions) and net costs of $6,460 in Nigeria, with a cost per DALY averted of $3.13. Results of providing the NASG for women in mild shock has smaller and uncertain effects due to few clinical events in this data set.

Conclusion: Using the NASG for women in severe shock resulted in markedly improved health outcomes (2–2.9 DALYs averted per woman, primarily due to reduced mortality), with net savings or extremely low cost per DALY averted. This suggests that in resource-limited settings, the NASG is a very cost-effective intervention for women in severe hypovolemic shock. The effects of the NASG for mild shock are less certain.

Citation: Sutherland T, Downing J, Miller S, Bishai DM, Butrick E, et al. (2013) Use of the Non-Pneumatic Anti-Shock Garment (NASG) for Life-Threatening Obstetric Hemorrhage: A Cost-Effectiveness Analysis in Egypt and Nigeria. PLoS ONE 8(4): e62282. doi:10.1371/journal.pone.0062282

Editor: Roger C. Young, University of Vermont College of Medicine, United States of America

Received October 13, 2012; **Accepted** March 19, 2013; **Published** April 30, 2013

Copyright: © 2013 Sutherland et al. This is an open-access article distributed under the terms of the Creative Commons Attribution License, which permits unrestricted use, distribution, and reproduction in any medium, provided the original author and source are credited.

Funding: The authors have no support or funding to report.

Competing Interests: The authors have declared that no competing interests exist.

* E-mail: janelle.downing@gmail.com

Introduction

The global health agenda has prioritized reduction of maternal mortality for the last two decades. Despite a worldwide decline of 35% from 1990 to 2008,[1] many countries will not meet Millennium Development Goal Five (MDG 5) of reducing maternal mortality by 75% before 2015. [2] The majority of maternal deaths occur in resource-limited settings, despite recent improved access to skilled attendance at delivery, maternity homes in rural areas, emergency obstetric care, mobile clinics, and preconception care.[3],[4],[5].

Obstetric hemorrhage is the most significant contributor to maternal mortality.[6],[7] Delays in identifying hemorrhage, reaching tertiary care facilities, and receiving definitive care such as blood transfusions and surgeries are factors that lead to maternal deaths in limited-resource settings. The non-pneumatic anti-shock garment (NASG) is a low-technology device that offers a potential solution to counteract the effect of these delays. Trials of the NASG in Egypt and Nigeria have demonstrated a 46%

decrease in blood loss, 45% decrease in mortality and 81% decrease in severe morbidity in women presenting with obstetric hemorrhage of all etiologies.[8].

Implementation of evidence-based maternal mortality interventions is limited by availability of resources and often depends on the strength of the health system.[9] Strategies that target women with intrapartum complications that can be managed with basic emergency obstetric care have been shown to be effective in reducing maternal mortality.[10] Interventions that can be used broadly within the health system have the most potential for making a large-scale impact. For example, use of misoprostol for community-based treatment of postpartum hemorrhage (PPH) is considered highly cost-effective, with an incremental cost per disability adjusted life year (DALY) of $6.[11].

However, no analysis has examined the cost-effectiveness of the NASG. Application of cost-effectiveness analyses is often limited by use of simulated data. If possible, clinical results are preferred in order to capture unexpected costs.[12] We conducted a cost-effectiveness analysis of the NASG using results from trials in several tertiary facilities in Egypt and Nigeria.

Materials and Methods

Overview

We assessed the cost-effectiveness of adding the NASG to standard hypovolemic shock management. We used clinical data from two published intervention trials, in Egypt and Nigeria, on the effects of the NASG added to standard care on maternal morbidity and mortality at tertiary facilities.[8] The analysis focused on a standardized group of 1,000 women presenting in shock, with the proportions of severe (mean arterial pressure [MAP] $\leq$60 mmHg) and mild (MAP>60 mmHg) shock specified by country to reflect patient characteristics in the clinical trials. We then examined three intervention scenarios: (a) no use of the NASG (i.e., standard care, as the reference case) for any woman in shock; (b) women in severe shock receive the NASG and women in mild shock receive standard care; and (c) all women in shock receive the NASG, regardless of initial shock status. Each scenario was compared incrementally to the reference group (No NASG for any woman in shock). Clinical outcomes (mortality and morbidities) were translated into disability adjusted life years (DALYs), and compared incrementally across scenarios. Costs for the NASG (materials, training, personnel) were collected from project records, and treatments for maternal hemorrhage (e.g., emergency hysterectomy, blood transfusion, and uterotonics) were obtained from local investigators and published sources. All costs were converted into 2010 international dollars. Net cost (or savings) for the NASG was calculated considering the sum of costs for NASG and for clinical management of maternal hemorrhage. Cost-effectiveness was calculated as the cost per DALY averted, which represents the cost for each unit of "disease burden" that the intervention prevents. We conducted a one-way sensitivity analysis. Methods are further described below and in the Technical appendix S1.

Clinical data

We obtained clinical information from two studies that used a two-phase design (pre-intervention (standard care) and NASG intervention plus standard care), conducted in four sites in Nigeria and two sites in Egypt. Funding for these studies came from The John D. and Catherine T. MacArthur Foundation. The pre-intervention phase employed standard care including etiology identification, fluid resuscitation, and uterotonic administration for those with uterine atony.[13] The intervention phase added the NASG. A total of 1,442 patients with obstetric hemorrhage of any etiology (ranging from ectopic pregnancy to ruptured uterus) were studied. The six participating facilities logged over 100,000 deliveries during the study period, with a severe hemorrhage rate of 1.4%. Women with MAP$\leq$60 had a significantly greater risk of morbidity and mortality and required additional care.[8] Severe morbidity was defined by the Mantel criteria as end-stage organ dysfunction.[14] Additional information is available in the original study manuscripts.[8,15,16].

The NASG (Zoex Corporation, Colma, CA) resembles the bottom half of a neoprene wetsuit with an abdominal foam compression ball and Velcro closures that allow perineal and abdominal access; circumferential pressure reduces blood flow to the pelvis and lower extremities and increases cardiac and cerebral perfusion (Figure 1). During the pre-intervention phase, women with hypovolemic shock secondary to obstetric hemorrhage from any etiology were treated with a standardized shock/hemorrhage protocol.[16] Their outcomes were compared to outcomes for similar women treated with the same protocol and the NASG during the intervention phase.

Variables selected for this analysis were in two categories: treatments and outcomes (Table 1). ***Treatment variables*** included mean uterotonic doses, mean units transfused blood, emergency hysterectomies for women with a primary diagnosis of uterine atony and hysterectomies for women with any obstetric hemorrhage etiology. ***Outcome variables*** included mean measured blood loss after drape placement, mean discharge hemoglobin and total number of women with severe anemia or hemoglobin<7.0 g/dl on discharge (approximated from hematocrit by dividing by a factor of 2.95, rounded to 3, for Nigeria[17]), severe morbidity and mortality. We omitted variables that did not differ significantly in the trials by NASG status including other surgeries and procedures.

Deaths were translated to DALYs using a value of 24 DALYs per death in Egypt and 22 per death in Nigeria. This was based on the difference between median age at maternal death, 28 and 30 and life expectancy at age 70 and 64 for Egypt and Nigeria respectively.[18] DALYs per other adverse event ranged from 0.09 for transient severe anemia to 2.5 for infertility secondary to emergency hysterectomy and 9 for long-term motor deficit (which occurred once, in Nigeria). Details on DALYs are provided in the Technical appendix S1.

Costs

We included treatment costs in both clinical study phases, as well as program costs in the intervention phase. The following equations were used to calculate costs:

$$(1)\ \text{Cost of standard of care} = [\text{Uterotonics}] + [\text{Blood transfusions}] + [\text{Emergency hysterectomy}]$$

$$(2)\ \text{Cost of NASG intervention} = [\text{Uterotonics}] + [\text{Blood transfusions}] + [\text{Emergency hysterectomy}] + [\text{Training}] + [\text{NASG}]$$

Cost categories included clinical material (disposable and reusable), facility, provider, and laboratory. Unit cost data were collected from local investigators at El Galaa Maternity Hospital in Cairo, Egypt, Assiut University Women's Health Center in Assiut, Egypt, University College Hospital in Ibadan, Nigeria and Katsina

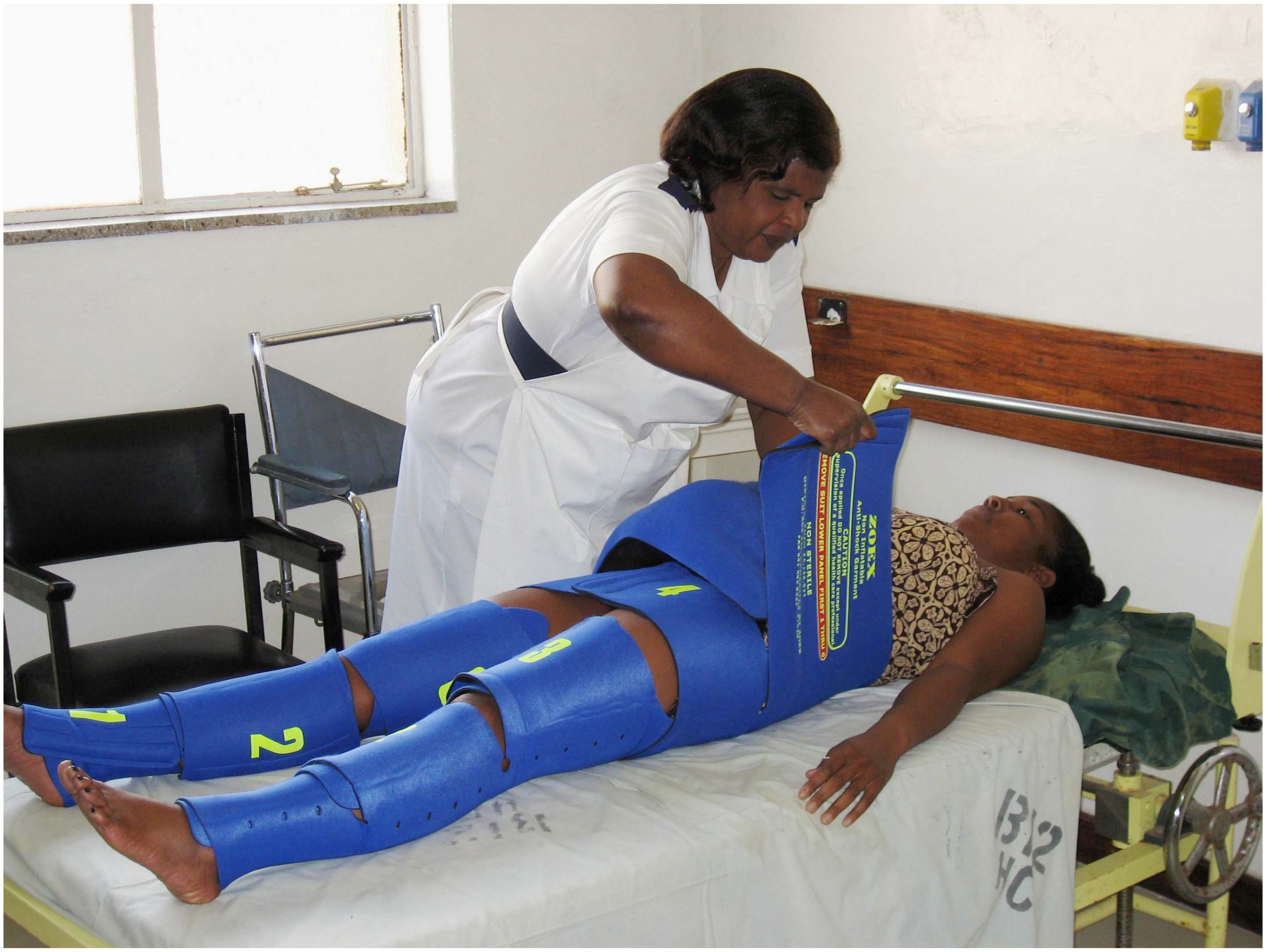

Figure 1. Image of nurse applying non-pneumatic shock garment (NASG) to study patient in Nigeria*. * Both the provider and patient have given informed consent, as outlined in the PLOS consent form, to publication of their photograph.
doi:10.1371/journal.pone.0062282.g001

General Hospital in Katsina, Nigeria (Table 2). Respondents reported using internal cost accounting systems and hospital charges to patients in order to estimate costs. Due to wide (30-fold) differences in uterotonic unit costs across the Nigerian hospitals, we used a conservative market price.[19] We examined uncertainty in unit costs in the sensitivity analyses.

Cost-effectiveness

We developed a cost-effectiveness model in Microsoft Excel 2004 to calculate net costs and DALYs from adding the NASG to a standard protocol for obstetric hemorrhage and hypovolemic shock. Calculations were performed for 1,000 women experiencing life-threatening obstetric hemorrhage, using standard economic methods, and reflecting the country-specific observed proportions of women in mild and severe shock (Table 3) [20]. Net costs (or savings) were calculated across intervention scenarios (no NASG for women in shock, NASG for severe shock only, and NASG for all women in shock), for each country (Table 4). Cost-effectiveness was calculated as net cost per DALY averted, or reported as "dominant" for intervention scenarios with lower costs and better health outcomes.

Training costs to treat 1,000 women were annualized assuming that training effects would last 10 years. Training components (venues, trainee per diems, and trainer salaries) and prices were estimated based on the experience in the Nigerian and Egyptian studies.

Intractable uterine atony is the only hemorrhage etiology for which emergency hysterectomy can be directly reduced by the NASG, because pelvic vascular compression can control blood loss until uterine contraction occurs. However, women who present with shock secondary to other obstetric hemorrhage etiologies (e.g., abruption placenta, ruptured uterus) and are stabilized with the NASG may then survive long enough to have the chance to receive the needed hysterectomy. For this reason, costs were examined in two ways: for hysterectomies secondary to primary uterine atony and for hysterectomies of all etiologies. Results were similar for both approaches, so we present results using the narrower scope and can provide the broader analysis on request.

All costs were converted from Egyptian Pounds and Nigerian Naira to 2010 international dollars using the most recently available conversation factors.[21]

We conducted sensitivity analyses to take into account possible variation in our baseline values. A one-way sensitivity analysis adjusted input parameters by 50% above or below the base case or used published ranges if available from prior NASG trials[8,22] (Table 5 and technical appendix S1). We also examined different

Table 1. Clinical trial results for women with shock from maternal hemorrhage, by country, shock severity, and use or non-use of the non-pneumatic anti-shock garment (NASG).

		Egypt		Nigeria	
		**No NASG**	**NASG**	**No NASG**	**NASG**
**1. Number of women in study trials, unstandardized**	**All**	432	558	175	277
	**Severe Shock**	72	106	109	215
**2. Mean units of blood transfused per patient**[1]	**All**	2.30	1.61	1.81	1.96
	**Severe Shock**	3.30	2.38	2.06	1.96
**3. Emergency hysterectomy, rate per 1,000** [**2**]	**All**	34.01	32.26	0	3.62
	**Severe Shock**	180.56	56.60	0	4.65
**4. Mean units of uterotonics given per patient (all)**[**3**]	**All**	2.01	1.71	2.59	1.58
	**Severe Shock**	2.70	1.65	2.65	1.68
**5. Proportion of women with discharge Hb<7.0 g/dl**	**All**	4.5%	4.9%	69.1%	61.5%
	**Severe Shock**	20.0%	12.2%	80.4%	66.2%
**6. Severe morbidity,%**	**All**	4.1%	0.9%	2.7%	0.4%
	**Severe Shock**	16.7%	3.1%	4.7%	0.0%
**7. Mortality,%**	**All**	2.3%	1.1%	16.0%	8.7%
	**Severe Shock**	12.5%	5.7%	22.0%	9.3%

[1]. 450 mls per unit [2]. Uterine atony only [3]. Oxytocin and Ergometrine.
doi:10.1371/journal.pone.0062282.t001

approaches to assigning unit costs in Nigeria due to the very different costs between Northern and Southern Nigeria.

Results

Clinical outcomes

The published trial data, adjusted to standard cohorts of 1,000 women, are displayed in Table 3 by country and intervention scenario; no NASG for women in shock, NASG for women in severe shock only, and NASG for all women in shock. The clinical effect of the NASG was most significant for women presenting with severe shock. Use of the NASG in this group reduced overall cohort mortality by 52% in both groups (13–91 per 1000 women in shock), and severe morbidities by 50–100% (24–34 per 1000 women in shock). Units of blood transfused decreased by 7% in Egypt and 4% in Nigeria. The number of units of uterotonics administered was roughly 9% lower in Egypt and 25% lower inNigeria.

When all women received the NASG in Egypt, there were directionally similar but much smaller magnitude and statistically

Table 2. Unit costs by study site, 2010 (Int$)[1].

**Costs**	**Egypt**			**Nigeria**		
	**El Galaa**	**Assiut**	**Average**	**UCH**	**Katsina**	**Average**
1. Hysterectomy[2]	514.72	51.47	283.09	831.03	75.40	453.21
2. 500 ml Blood transfusion	77.21	51.47	64.34	83.10	32.78	57.94
3. 10iu Oxytocin	1.12	1.16	1.14	1.11[3]	0.41	0.76
4. 0.2 mg Ergometrol	1.02	1.02	1.02	4.16	0.27	2.21
5. Training cost per patient[4]	5.27	5.27	5.27	3.57	3.57	3.57
6. NASG per use[5]	7.39	7.38	7.38	7.79	7.79	7.79

[1]. Costs were adjusted from local currency to international dollar with most recently available purchasing power parity (PPP) factors of 2 for Egypt and 78 for Nigeria.[21] Refer to Technical appendix S1 for a detailed explanation.
[2]. Differences in hysterectomy cost were investigated and confirmed with local investigators.
[3]. Oxytocin cost from PATH report[28] for price per dose of $0.55 USD for an occasional purchase, medium volume.
[4]. Training costs were standardized across project sites and include provider time during the training.
[5]. Cost includes purchase price of $295 USD based on 40 uses.
doi:10.1371/journal.pone.0062282.t002

Table 3. Treatments and outcomes for standardized cohorts of 1,000 women with shock from maternal hemorrhage, by NASG intervention scenario[1,2].

	Egypt			Nigeria		
**Intervention scenario:**	**No NASG**	**NASG only if MAP<60**	**NASG for all**	**No NASG**	**NASG only if MAP<60**	**NASG for all**
**1. Projected total blood transfusion for 1,000 women, mls**	1,045,821	971,435	722,024	843,555	812,981	880,606
**2. Emergency hysterectomies**						
**Emergency hysterectomy-uterine atony dx only, n**	37	15	32	0	3	3
**All emergency hysterectomies**	95	68	54	0	17	21
**3. Uterotonic (any)**						
**Oxytocin, total 10iu doses**	1,433	1,303	1,226	2,401	1,793	1,499
**Ergometrine, total.2 mg doses**	578	520	488	191	99	80
**4. Total number of women with discharge Hb<7.0 g/dl**	59	45	53	739	637	626
**5. Severe morbidity, n**	48	24	9	34	0	5
**6. Mortality, n**	25	12	10	175	84	85

[1]. Proportion of cohort with severe shock (MAP<60) is 18% for Egypt and 72% for Nigeria. This is derived from clinical trial data[15,16], combining pre and post trial periods.
[2]. Top 3 definitive diagnoses by country and study phase: Egypt: [Pre (N = 432): Uterine atony (34%), Ectopic pregnancy (19%), Placental abruption (14%)] [Post (N = 558): Uterine atony (44%), Abortion complications (14%), Ectopic pregnancy (13%)] Nigeria [Pre (N = 175): Retained placenta (30%), Uterine atony (25%), Placental abruption (11%)] [Post (N = 277): (Uterine atony (26%), Retained placenta (23%), Placental abruption (17%)].
doi:10.1371/journal.pone.0062282.t003

non-significant benefits. In Nigeria, blood transfusions increased by 8.3%, and there were small and statistically non-significant increases in mortality and severe morbidity.

In Egypt, the number of hysterectomies among women with uterine atony was 59% lower if women in severe shock receive the NASG. In Nigeria, there were very few hysterectomies performed; all were in the NASG phase and most were not associated with uterine atony. The reported number of women in severe shock with severe anemia (Hb<7.0) on discharge was lower for women using the NASG, in both Egypt and Nigeria.

These clinical findings translate into 357 DALYs averted by NASG use for women in severe shock in Egypt and 2063 in Nigeria (Table 4). Reduced mortality accounts for most (82–96%) of the DALYs averted in this cohort (not in table). If women in mild shock also received the NASG, the estimates of incremental changes in DALYs (+27 to –37) are much smaller and unstable

Table 4. Calculated mortality, morbidity, disability-adjusted life years, cost, and cost-effectiveness for 1,000 women with severe or mild shock[1] from maternal hemorrhage, by intervention scenario.

Intervention scenario	Mortality		Morbidity		Total DALYs		Cost		ICER ($ per DALY averted)[3]	
	# per 1000	Difference[2]	# per 1000	Difference	# per 1000	Difference	$ per 1000	Difference	Incremental	vs. No NASG
**Egypt**										
**No NASG**	24.7		48		730		147,248			
**NASG only if MAP < 60**	12.4	−49.79%	24	−50.59%	372	−357	137,760	−9,489	Dominant	
**NASG for all**[4]	10.2	−17.93%	9	−159.39%	335	−37	116,507	−21,253	Dominant	Dominant
**Nigeria**										
**No NASG**	175.0		34		3960		100,003			
**NASG only if MAP < 60**	83.8	−52.09%	0	−100.00%	1896	−2063	106,463	6,460	$3.13	
**NASG for all**[4]	85.2	1.68%	5	N/A	1923	27	116,240	9,777	Dominated	$7.97

1. Proportion of cohort with severe shock (MAP<60) is 18% for Egypt[16] and 72%[15] for Nigeria (from clinical trial, combining pre and post trial periods).
2."Difference" is versus prior row (scenario). Negative values in "difference" columns are desirable — disease burden or cost averted.
3. "Dominant" means cheaper and better health outcomes than previous intervention scenario. "Dominated" means more costly and worse outcomes than previous scenario.
4. Results for "NASG only if MAP <60" are more stable than for the incremental effect of "NASG for all", due to the small number of clinical events for mild shock group in original trials; see text.
doi:10.1371/journal.pone.0062282.t004

Table 5. One-way sensitivity analysis on cost and health differences between two intervention scenarios: NASG for severe shock and No NASG.

	Egypt			Nigeria		
	Input values		Results: Difference between NASG for severe shock and No NASG	Input values		Results: Difference between NASG for severe shock and No NASG
Input	Base case	Lower and upper bound		Base case	Lower and upper bound	
**Costs**			Net $ per 1000 women with shock			Net $ per 1000 women with shock
Base case result			−$9,489			$6,460
1. NASG (per use)	$*7.38*	1.36[1], 11.06	−10,572, −8,828	$*7.79*	3.9[1], 11.7	3,671, 9,262
2. Uterotonics	$*1.14*	0.57, 1.71	−9,388, −9,603	$*0.76*	0.38, 1.14	6,327, 6,858
3. Blood transfusion	$*64.34*	32.17, 96.51	−4,703, −14,275	$*58*	29, 87	8,231, 4,688
4. Emerg. Hyst	$*283.09*	141.55, 424.64	−6,334, −12,643	$*453*	227, 680	5,704, 7,215
**Health**			Change in DALYs per 1000 women with shock			Change in DALYs per 1000 women with shock
Base case result			−357			−2,063
1. Mortality effect[2]	*1.00*	0.80, 1.2	−406, −309	*1.00*	0.80, 1.2	−2,354, −1,772
2. Morbidity effect	*1.00*	0.80, 1.2	−364, −351	*1.00*	0.80, 1.2	−2,074, −2,053
3. Severe anemia effect	*1.00*	0.50, 1.5	−358, −356	*1.00*	0.50, 1.5	−2,085, −2,041
4. Infertility	*1.00*	0.50, 1.5	−371, −344	*1.00*	0.50, 1.5	−2,068, −2059

[1]. Price based on cost provided by Blue Fuzion based in Hong Kong.
[2]. Health effect indicates risk of event compared with the base case, i.e., effect = 0.80 means 20% lower risk of mortality or morbidity for the "NASG for severe shock" scenario than assumed in the main analysis.
doi:10.1371/journal.pone.0062282.t005

due to few clinical events and thus statistical uncertainty in the original studies among women in mild shock.

Costs

The categories of cost include emergency hysterectomies, blood transfusions, uterotonics, and provider training for use of the NASG, and the NASG itself.

In Egypt, per 1,000 women, providing the NASG to women in severe shock resulted in net savings of Intl$ 9,489. Major savings were derived from reduced blood transfusions ($9,572). The primary added cost in this group was for the NASG and associated training. In Nigeria, using the NASG for severe shock resulted in incremental net costs of Intl $6,460. The NASG saved $3,543in blood transfusions. However, the savings were more than offset by higher costs of more hysterectomies, as well as the costs of the NASG and training. Providing the NASG for all women in shock resulted in further savings in Egypt and further costs in Nigeria, though again with uncertainty due to precision issues.

Cost-effectiveness

Providing the NASG to women in severe shock is cost-saving for Egypt and cost-effective for Nigeria. Cost-effectiveness ratios for women experiencing shock from maternal hemorrhage are summarized in the final columns of Table 4.

In Egypt, providing the NASG for women presenting with severe shock was "dominant," saving $9,489 and averting 357 DALYs per 1,000 women. (No cost-effectiveness ratio is needed with lower costs and better outcomes.) The benefits for treating all women with the NASG were lower (37 averted DALYs with $21,253 in incremental savings), and uncertain due to unstable estimates of clinical outcomes and thus DALYs, as noted above.

In Nigeria, with only women with severe shock receiving the NASG, the NASG had a net cost of $6,460 and averted 2,063 DALYs, for an incremental cost-effectiveness ratio (ICER) of Intl$ 3.13 per DALY averted. Unfavorable incremental results occur when women in mild shock also received the NASG: $9,777 in increased costs and 27 additional DALYs. As with the small gains in Egypt, these results are uncertain due to unstable estimates of clinical outcomes and thus DALYs.

In settings where the intermediate intervention scenario (NASG only for women in severe shock) is considered infeasible, it is appropriate to compare the more extreme scenarios. In Egypt, "NASG for all" option dominates "No NASG". In Nigeria, this comparison has a cost-effectiveness ratio of $4.13 per DALY averted.

Sensitivity analyses

One-way cost-effectiveness analyses for the uterine atony-only hysterectomy scenario with women in severe shock only receiving the NASG are presented in Table 5. Per standard protocol, costs were varied +/− 50% unless market cost ranges were available (i.e. NASG $1.36–11.06 in Egypt; $3.90–11.70 in Nigeria; single use). As the cost of blood transfusion increases, the NASG becomes more cost saving in Egypt. Varying the cost of blood by +/− 50%, we found overall cost savings in Egypt ranged from $4,703–14,275, while in Nigeria the net cost ranged from $4,688–8,231. Even when the number of units of blood was removed from the equation, the NASG remained cost saving and cost-effective in Egypt and Nigeria, respectively. Removing uterotonics from the equation had a minor effect on the cost-effectiveness.

Although the clinical trials used garments purchased at $170 USD, the current (higher) market value of $295 was used in this analysis. When we increased the cost of the NASG at baseline by 50% to $11.06 ($11.70 in Nigeria) per use in the severe shock group, the cost savings in Egypt fell by roughly 7%. When we set the price of the NASG at the lowest available market cost [SM1] of $53.76 plus cleaning, or $1.36 per use in Egypt and $3.90 in Nigeria, savings in Egypt increased by 10% and costs in Nigeria decreased by 43%.

The DALYs health effect estimates for women in severe shock receiving the NASG (+/– 20% for morbidity and mortality; +/– 50% for severe anemia and infertility) are most sensitive to mortality parameters. As the NASG increases its efficacy in saving lives, more DALYs are averted. For example, when the NASG is 20% more effective at mortality reduction, women in severe shock gain an additional 49 DALYs in Egypt and 291 DALYs in Nigeria. Altering the effect of severe morbidity, severe anemia and infertility have less significant impact on total DALYs.

Discussion

For women in severe shock, the NASG intervention, even with conservative assumptions (highest price), is an economically attractive option for health systems aiming to reduce maternal mortality from hemorrhage and shock. It is cost-saving in Egypt, when compared to standard care only. In Nigeria, it is highly cost-effective, $3 per DALY averted, far below the WHO standard for "very cost-effective" defined as the annual gross domestic product (GDP) per capita.[23] In comparison, a highly favorable maternal mortality intervention in urban India focused on improved intrapartum care had costs of $150–350 per DALY averted.[24]

Importantly, the NASG provides a very high health benefit for women in severe shock. For each woman in severe shock receiving the garment, the average expected benefit is 2.0 – 2.9 DALYs averted, i.e., between 2 and 3 added years of life. Few interventions provide this magnitude of health gain. The results with mild shock are of far lower magnitude, and uncertain in direction due to small numbers of events (especially deaths) and thus statistical uncertainty in the clinical trials.

The financial benefit of the NASG is that it allows providers to use fewer medical care resources. In some low-income settings, safe units of blood are scarce and often sold on the black market for high prices. In Nigeria, like other developing countries, patients may die because of deficiencies in blood banking or lack of supply.[25] The cost of blood in our analysis is roughly 60 international dollars per unit. In Egypt, patients with the NASG use, on average, 0.8 fewer units of blood than those without. In Nigeria, blood supply is highly variable: from the authors' clinical experience, patients often receive blood only if a family member donates it, and thus its use may be more contingent on availability than clinical need. Yet the average number of units of blood was slightly lower with the NASG for women in severe shock.

Likewise, women in severe shock who were put in the NASG used an average of one fewer dose of an uterotonic, in both Egypt and Nigeria. While the cost of a dose of uterotonics is significantly less than a unit of blood, each provide an example of how the use of the NASG can conserve resources. Additional research is needed to determine the contexts within and to the degree to which the NASG is effective in reducing use of blood, uterotonics, and number of surgeries.

In this analysis, emergency hysterectomies served as an indicator for the severity of a woman's hemorrhage; if the hemorrhage was uncontrolled, or appeared uncontrollable, by medical or more conservative surgeries, a hysterectomy might be performed as an extreme effort to save the woman's life. In previous trials,[15,16] the efficacy of the NASG was most definitive for women with a diagnosis of uterine atony. Often for women with other diagnoses, such as placental abruption, an emergency hysterectomy still may be required despite the efficacy of the NASG in controlling bleeding; indeed, the NASG may stabilize women enough to permit life-saving hysterectomies. For women in severe shock, hysterectomies decreased with the NASG for both uterine atony and all etiologies in Egypt, thus saving resources. In Nigeria, the rate of hysterectomies overall was very low (2%), and in the trial only one hysterectomy was performed for a woman with uterine atony who received the NASG. Hysterectomies for other etiologies rose with NASG. Additional studies are needed to determine the efficacy of the NASG in conserving surgical resources across various settings, and if the increase in surgeries was due to the increased chance of a woman surviving long enough to receive the surgery if she had the NASG compared to those who did not.

If the savings due to the averted use of medical resources is excluded, the "crude" ICER for the NASG material costs and training is Intl$ 6.37 in Egypt and 3.95 in Nigeria (for the analysis including hysterectomies for uterine atony only). This means that use of the NASG appears very cost-effective even in a clinical setting where blood, uterotonics, and surgery are unavailable. Additional studies are needed to determine the cost-effectiveness of the NASG in a variety of clinical settings to set priorities for implementation.

This analysis was stratified by shock level because resource allocation and protocol for shock management is often dependent on severity of shock. The NASG is very favorable for women with severe shock, but has far smaller and, in this study, inconsistent results for those with mild shock. In Egypt, there was only one death in the entire study (N = 619) for those in mild shock. As mortality accounts for a majority of DALYs averted in the severe shock phase, the mild shock results are markedly different, with a high proportion of DALYs contributed by the infertility associated with hysterectomies.

In Nigeria, the number of women in the trial's mild shock group (N = 127 total pre- and with NASG) was markedly smaller than for the other groups. Nigerian women in these studies had less access to care compared to women in the Egyptian study sites, and were more likely to come to the facility already bleeding and in severe shock. Thus, the standardized cohort size of 1000 exaggerated the number of key clinical events. There were 4 deaths out of 66 without the NASG, and 4 deaths out of 61 with the NASG, suggesting a statistically insignificantly higher mortality in the NASG group. Also, there was only one hysterectomy. Therefore, DALY calculations are highly uncertain. The number of units of blood and doses of uterotonics also increased, for unclear reasons. Perhaps these changes were due to temporal changes associated with better adherence to protocol, or may reflect changes due to availability of resources. More research is needed to determine the cost-effectiveness of the NASG for women presenting in mild shock.

NASG use resulted in reductions in severe morbidity, ranging from 81–100% for women presenting with severe shock. Cost estimates are conservative and exclude cost offsets from a decline in anemia. The NASG reduced the number of women with severe anemia (by 39% in Egypt and 18% in Nigeria). Although we could not measure the effect of anemia on the study participants' postpartum productivity, anemia has been associated with decreased postpartum economic productivity (Galloway 2003[26]).

The range of trial results by site and country indicate that the NASG will have varied effects on clinical practice, morbidity, and mortality, dependent on pre-existing resources and patient condition on arrival. A large-scale, randomized cluster trial currently being conducted in Zambia and Zimbabwe may shed more light on the effect of the NASG in the context of different health systems.

A limitation of this analysis is the variability in cost collection methods from local investigators. Some sites were only able to report hospital fees for emergency hysterectomy because they had no access to complete cost data. The collected costs were compared to costs detailed in the CHOICE series,[23] which were lower than costs at UCH and El Galaa and similar to the other two sites. A sensitivity analysis was performed in order to more accurately represent health system costs.

Scaling up the NASG by distributing a larger number to each health system region may provide opportunities for greater efficiency. An identical NASG prototype has been developed and tested for $53.76 per garment as opposed to the current price of $295[27] per garment, and can be purchased in a minimum order of 1,000 garments (Blue Fuzion, Hong Kong[28]). Additional manufacturers are being added in Asia. The NGO organization PATH has recommended establishment of regional distributors, and of exclusive garment rights, if necessary to protect from excessively high prices.[28] Additionally, if training on the NASG were included in the standard emergency obstetric care curriculum for medical, midwifery and nursing students, programmatic training costs would be reduced.

We found that the NASG is cost saving or highly cost-effective for women in severe hypovolemic shock when administered in a tertiary care setting. It may become even less costly to implement if sales prices continue to decrease, and it becomes part of pre-service training. Future research is needed to validate the cost-effectiveness and efficacy of the NASG in other settings. The Zambia and Zimbabwe trial noted above compares effect on outcomes of earlier application of the NASG at the community level before transport to tertiary facilities. Once those results are published, we will conduct a CEA of NASG use to determine level of care at which the NASG will be most cost-effective.

Supporting Information

Technical Appendix S1 Description of methods for collecting input data, calculating cost-effectiveness, and performing sensitivity analyses.
(DOC)

Author Contributions

Conceived and designed the experiments: SM TS. Performed the experiments: TS JD. Analyzed the data: JD TS. Contributed reagents/materials/analysis tools: EB DMB MF MMY OO DN JGK. Wrote the paper: JD TS JGK. Designed and implemented the analysis, and drafted the manuscript: TS. Prepared the health and cost input data, refined the analysis, and edited the manuscript: JD. Conceptualized the project, commented on analyses, and edited drafts: SM. Provided conceptual guidance: DMB. Gathered health input data, and commented on input analysis and drafts: EB. Provided estimates of costs from clinical sites, and approved the final manuscript: MF MMY OO DN. Supervised the analysis and edited the manuscript: JGK.

References

1. Hogan MC, Foreman KJ, Naghavi M, Ahn SY, Wang M, et al. (2010) Maternal mortality for 181 countries, 1980–2008: a systematic analysis of progress towards Millennium Development Goal 5. Lancet 375: 1609–1623.
2. United Nations. (2011) Millenium Development Goals Indicators: Purchasing power parties (PPP) conversion factor, local currency unit to international dollar.
3. Kidney E, Winter HR, Khan KS, Gulmezoglu AM, Meads CA, et al. (2009) Systematic review of effect of community-level interventions to reduce maternal mortality. BMC Pregnancy Childbirth 9: 2.
4. UNICEF (2010) Goal 5: Improve Maternal Health; Fact Sheet. New York.
5. Atrash HK, Johnson K, Adams M, Cordero JF, Howse J (2006) Preconception care for improving perinatal outcomes: the time to act. Matern Child Health J 10: S3–11.
6. Khan KS, Wojdyla D, Say L, Gulmezoglu AM, Van Look PF (2006) WHO analysis of causes of maternal death: a systematic review. Lancet 367: 1066–1074.
7. Ordi J, Ismail MR, Carrilho C, Romagosa C, Osman N, et al. (2009) Clinico-pathological discrepancies in the diagnosis of causes of maternal death in sub-Saharan Africa: retrospective analysis. PLoS Med 6: e1000036.
8. Miller S, Fathalla MM, Ojengbede OA, Camlin C, Mourad-Youssif M, et al. (2010) Obstetric hemorrhage and shock management: using the low technology Non-pneumatic Anti-Shock Garment in Nigerian and Egyptian tertiary care facilities. BMC Pregnancy Childbirth 10: 64.
9. Nyamtema AS, Urassa DP, van Roosmalen J (2011) Maternal health interventions in resource limited countries: a systematic review of packages, impacts and factors for change. BMC Pregnancy Childbirth 11: 30.
10. Campbell OM, Graham WJ (2006) Strategies for reducing maternal mortality: getting on with what works. Lancet 368: 1284–1299.
11. Sutherland T, Meyer C, Bishai DM, Geller S, Miller S (2010) Community-based distribution of misoprostol for treatment or prevention of postpartum hemorrhage: cost-effectiveness, mortality, and morbidity reduction analysis. Int J Gynaecol Obstet 108: 289–294.
12. American College of Physicians (ACP): Internal Medicine. Primer on Cost-Effectiveness Analysis. Effective Clinical Practice SOAoSaahw.
13. Gulmezoglu AM, Souza JP, Chou D, Mathai M, Hill S, et al. (2009) WHO guidelines for the management of postpartum haemorrhage and retained placenta. Geneva.
14. Mantel GD, Buchmann E, Rees H, Pattinson RC (1998) Severe acute maternal morbidity: a pilot study of a definition for a near-miss. Br J Obstet Gynaecol 105: 985–990.
15. Miller S, Ojengbede O, Turan JM, Morhason-Bello IO, Martin HB, et al. (2009) A comparative study of the non-pneumatic anti-shock garment for the treatment of obstetric hemorrhage in Nigeria. Int J Gynaecol Obstet 107: 121–125.
16. Miller S, Fathalla MM, Youssif MM, Turan J, Camlin C, et al. (2010) A comparative study of the non-pneumatic anti-shock garment for the treatment of obstetric hemorrhage in Egypt. Int J Gynaecol Obstet 109: 20–24.
17. Van Slyke D, Phillips RA, Dole VP, Hamilton P, Archibald RM, et al. (1950) Calculation of Hemoglobin from Blood Specific Gravities. Journal of Biological Chemistry 183: 331–347.
18. (2006) Disease control priorities in developing countries, 2nd Ed. Jamison D, Breman JG, Measham AR, Alleyne G, Claeson M, Evans DB, Jha P, Mills A, Musgrove P, editors. Washington DC: Oxford University Press
19. Seligman BL, Liu X (2006) Economic assessment of interventions for reducing postpartum hemorrhage in developing countries. Bethesda: Abt.
20. Drummond M OBB, Stoddart G, Torrance G (1997) Methods for the Economic Evaluation of Health Care Programmes, Second Edition. London: Oxford University Press.
21. (2011) PPP conversion factor, GDP. World Bank.
22. Krahn MD, Naglie G, Naimark D, Redelmeier DA, Detsky AS (1997) Primer on medical decision analysis: Part 4—Analyzing the model and interpreting the results. Med Decis Making 17: 142–151.
23. Acharya A, Adam T, Baltussen R, Evans D, Hutubessy R, et al. (2003) Making Choices in Health: WHO Guide to Cost Effectiveness Analysis. Geneva: World Health Organization. 250 p.
24. Goldie SJ, Sweet S, Carvalho N, Natchu UC, Hu D (2010) Alternative strategies to reduce maternal mortality in India: a cost-effectiveness analysis. PLoS Med 7: e1000264.
25. Osobu-Asubiojo FBR (2011) Merchants of Blood. The News of Africa.
26. Galloway R (2003) Anemia prevention and control - What works? Part 1 | Part 2 USAID, World Bank, PAHO/WHO, Micronutrient Initiative, FAO, and UNICEF.
27. World Health Organization. (2011) Compendium of new and emerging health technologies.
28. PATH (2011) Affordable, High-Quality, Non-Pneumatic Antishock Garments. Seattle: PATH. 1–85 p.
